# GRAS-1 is a novel regulator of early meiotic chromosome dynamics in *C*. *elegans*

**DOI:** 10.1371/journal.pgen.1010666

**Published:** 2023-02-21

**Authors:** Marina Martinez-Garcia, Pedro Robles Naharro, Marnie W. Skinner, Kerstin A. Baran, Laura I. Lascarez-Lagunas, Saravanapriah Nadarajan, Nara Shin, Carlos G. Silva-García, Takamune T. Saito, Sara Beese-Sims, Brianna N. Diaz-Pacheco, Elizaveta Berson, Ana B. Castañer, Sarai Pacheco, Enrique Martinez-Perez, Philip W. Jordan, Monica P. Colaiácovo

**Affiliations:** 1 Department of Genetics, Blavatnik Institute, Harvard Medical School, Boston, Massachusetts, United States of America; 2 Biochemistry and Molecular Biology Department, John Hopkins University, Bloomberg School of Public Health, Baltimore, Maryland, United States of America; 3 Department of Biochemistry and Molecular Biology, Uniformed Services University of the Health Sciences, Bethesda, Maryland, United States of America; 4 Department of Molecular Metabolism, Harvard T. H. Chan School of Public Health, Harvard University, Boston, Massachusetts, United States of America; 5 MRC London Institute of Medical Sciences, London, United Kingdom; Department of Cell and Developmental Biology, Biocenter, University of Wurzburg, GERMANY

## Abstract

Chromosome movements and licensing of synapsis must be tightly regulated during early meiosis to ensure accurate chromosome segregation and avoid aneuploidy, although how these steps are coordinated is not fully understood. Here we show that GRAS-1, the worm homolog of mammalian GRASP/Tamalin and CYTIP, coordinates early meiotic events with cytoskeletal forces outside the nucleus. GRAS-1 localizes close to the nuclear envelope (NE) in early prophase I and interacts with NE and cytoskeleton proteins. Delayed homologous chromosome pairing, synaptonemal complex (SC) assembly, and DNA double-strand break repair progression are partially rescued by the expression of human CYTIP in *gras-1* mutants, supporting functional conservation. However, *Tamalin*, *Cytip* double knockout mice do not exhibit obvious fertility or meiotic defects, suggesting evolutionary differences between mammals. *gras-1* mutants show accelerated chromosome movement during early prophase I, implicating GRAS-1 in regulating chromosome dynamics. GRAS-1-mediated regulation of chromosome movement is DHC-1-dependent, placing it acting within the LINC-controlled pathway, and depends on GRAS-1 phosphorylation at a C-terminal S/T cluster. We propose that GRAS-1 coordinates the early steps of homology search and licensing of SC assembly by regulating the pace of chromosome movement in early prophase I.

## Author summary

Successful sexual reproduction depends on the formation of gametes (i.e., eggs and sperm) carrying a correct number of chromosomes. This involves a series of well-choreographed steps during meiosis, the specialized cell division program resulting in the formation of eggs and sperm. Some of these steps include pairing between homologous chromosomes, assembly of a scaffold (the synaptonemal complex) between homologs, and inter-homolog recombination. These steps must be properly coordinated to ensure normal meiotic progression. Here, we show that the GRAS-1 protein in the nematode *C*. *elegans*, which shares sequence conservation with the mammalian proteins GRASP/Tamalin and CYTIP, contributes to normal meiotic progression by coordinating early events in meiosis. GRAS-1 is required for the normal speed of chromosome movements early in prophase I in a manner dependent of its phosphorylation and on dynein heavy chain. Delayed homologous chromosome pairing, synaptonemal complex assembly, and DNA double-strand break repair progression are partially rescued by the expression of human CYTIP in *gras-1* mutants, supporting functional conservation. We identified a new factor coordinating the early steps of pairing and synapsis by regulating the pace of chromosome movement in early prophase I.

## Introduction

Meiosis is a specialized cell division process in which diploid germ cells give rise to haploid gametes (i.e., eggs and sperm) accomplished by following a single round of DNA replication with two consecutive rounds of chromosome segregation. To segregate properly, homologous chromosomes must undergo a series of steps that are unique to the first meiotic division and are conserved from yeast to mammals, including: (1) pairing, (2) assembly of the “zipper-like” synaptonemal complex (SC) between paired homologs, and (3) formation of programmed meiotic DNA double-strand breaks (DSBs) resulting in crossover recombination, leading to genetic diversity and physical attachments between homologs [1]. Errors in any of these steps can result in impaired chromosome segregation and aneuploidy, which is associated with 20% of birth defects (e.g., Down Syndrome), 35% of clinically recognized miscarriages, infertility, and tumorigenesis [2].

During pairing, homologous chromosomes must physically align along their lengths; this is achieved by pronounced chromosome movements inside the meiotic nucleus driven by cytoskeletal forces in the cytoplasm. Cytoskeletal forces generated by dynein and microtubules in mammals, fission yeast, and worms, and by actin in budding yeast and plants, are transmitted through the nuclear envelope (NE)-spanning LINC (linker of nucleoskeleton and cytoskeleton) complex to chromosome ends tethered to the NE [3,4]. LINC complexes driving chromosome movements during meiosis consist of KASH domain proteins that span the outer nuclear membrane connecting the cytoskeleton to SUN domain proteins that span the inner nuclear membrane and extend into the nucleus where they can interact with chromosome ends via attachment proteins such as telomeric proteins [4,5]. In yeast, for example, KASH domain protein Csm4 transmits actin forces to the SUN domain protein Mps3, which in turn is connected by the adaptor protein Ndj1 to telomeres to produce the needed chromosome movements during leptotene and zygotene that ensure pairing between homologs [6–8]. In *C*. *elegans*, the meiotic LINC complex is formed by the KASH domain protein ZYG-12 at the outer nuclear membrane and the SUN domain protein SUN-1 at the inner nuclear membrane [9]. PLK-2-dependent phosphorylation of SUN-1 results in the formation of SUN-1 aggregates which interact via yet unidentified factors with one end of each chromosome carrying specific repetitive sequences (pairing centers, PCs) which are bound by PC end Zinc-finger proteins [10–12]. PC proteins facilitate chromosome movement until homologs begin pairing and assembling the SC [13]. The SC is a tripartite structure composed of proteins assembled along chromosome axes (lateral elements) and proteins that bridge each pair of axes (central region components) [14]. Studies in budding yeast, plants, flies, worms, and mammals, have shown that the SC is critical for stabilizing homologous chromosome pairing, the progression of meiotic recombination, crossover formation, and achieving accurate meiotic chromosome segregation [15]. Work in *C*. *elegans* has identified proteins involved in regulating pairing and SC formation [16–20], but how NE-associated proteins regulate chromosome dynamics during early prophase I is incompletely understood. Here we show that *C*. *elegans* GRAS-1, which is homologous to mammalian GRASP/Tamalin and CYTIP, localizes to the NE and is required for the regulation of chromosome movement. GRAS-1 limits the speed of dynein-microtubule driving forces and contributes to the licensing of SC assembly, ensuring adequate timing of key meiotic processes such as homologous chromosome pairing, SC assembly, and DSB repair progression. While mice *Tamalin*, *Cytip* double knockout (DKO) mutants did not display obvious SC and recombination defects, human CYTIP partially rescued a *gras-1* mutation, supporting functional conservation and suggesting evolutionary differences between the mammalian proteins. We propose a model by which GRAS-1 links NE-cytoskeleton-SC assembly and coordinates early meiotic events by acting as a brake during early meiotic prophase I chromosome movements.

## Results

### GRAS-1 localization is meiosis-specific and contacts NE components

**Fig 1:**
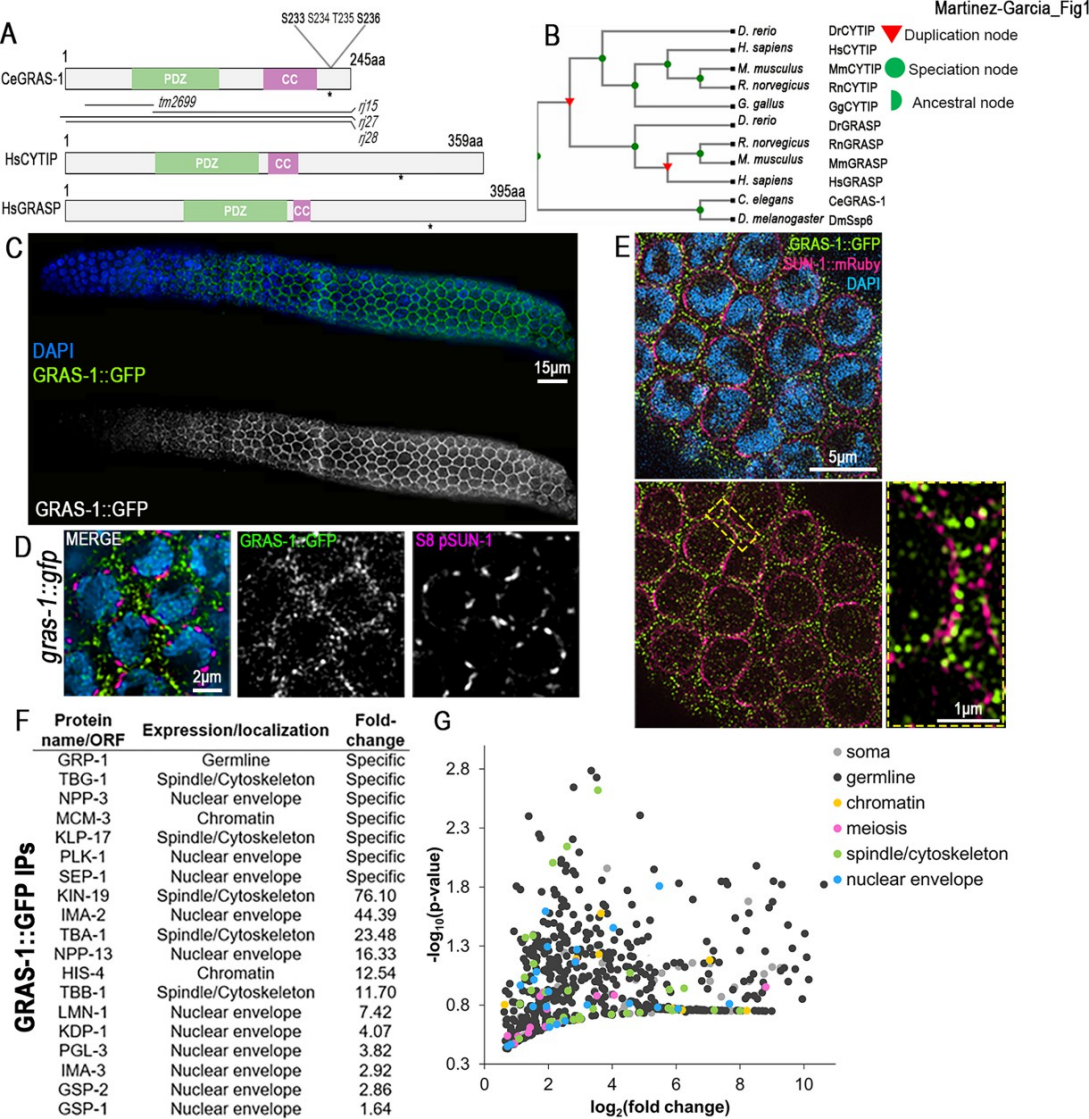
GRAS-1 localization is meiosis-specific and contacts NE components. **(A**) Protein conservation between *C*. *elegans* (Ce) GRAS-1 and *Homo sapiens* (Hs) CYTIP and GRASP. PDZ: PSD-95/SAP90, Discs-large and ZO-1, CC: coiled-coil, asterisk indicates position of predicted phosphorylation sites. **(B)** Evolutionary tree (TreeFam) of GRAS-1 orthologs in *Drosophila melanogaster* (Dm), *Mus musculus* (Mm), *Rattus norvegicus* (Rn), *Danio rerio* (Dr), *Gallus* (Gg), humans and worms. **(C)** GRAS-1::GFP localization in hermaphrodite gonads co-stained with anti-GFP (green) and DAPI (blue). **(D)** Higher magnification images of leptotene/zygotene stage nuclei co-stained with anti-GFP for GRAS-1::GFP (green), anti-S8 pSUN-1 (magenta) and DAPI (blue). **(E)** Super-resolution microscopy image of *gras-1*::*gfp* leptotene/zygotene nuclei co-stained for GRAS-1::GFP (green), SUN-1::mRuby (magenta) and DAPI. Dashed rectangle indicates region of the nuclear margins shown at higher magnification. **(F)** GRAS-1 interacting proteins. Immunoprecipitation from GRAS-1::GFP whole worm extracts was analyzed by mass spectrometry analysis. Their localization and enrichment in the MS samples compared to controls are shown. **(G)** Volcano plot depicting all MS analysis hits above a 1.5-fold change in GRAS-1::GFP samples compared to controls (x axis), their statistical significance (y axis) and colored by their described expression/localization in *C*. *elegans*.

A yeast-two hybrid screen for candidates interacting with worm SC proteins identified GRAS-1 [21]. *gras-1* (ORF F30F8.3) encodes for a 245 amino acid protein containing PDZ (PSD-95/SAP90, Discs-large, and ZO-1) and coiled-coil (CC) domains (Fig 1A). GRAS-1 shares a high degree of conservation with both human CYTIP (Cytohesin-interacting protein, 63% homology) and GRASP/Tamalin (General receptor for phosphoinositides 1-associated scaffold protein, 61% homology), due to a gene duplication event in chordates (Fig 1A and 1B, TreeFam). No orthologs were found in fungi or plants. GRASP has been implicated as a scaffold for multi-protein complexes involved in processes such as epithelial cell migration and membrane trafficking [22,23]. CYTIP plays roles in cell adhesion and the immune system [24]. In mice and humans, both GRASP and CYTIP are expressed in testes and ovaries (S1A Fig) [25,26]. In worms, *gras-1* exhibits germline-enriched expression that is restricted to meiosis by the RNA-binding protein PUF-8 (S1B Fig) [27–31]. However, the meiotic functions for GRAS-1 and its homologs remained unknown.

Different databases place GRAS-1 and its mammalian homologs at the plasma membrane, cytosol, membrane systems and the perinuclear region (WolFSORT, UniProt, Human Protein Atlas). The expression of a functional GRAS-1::GFP transgene is detected only in the germline where GRAS-1 exhibits a meiosis-specific localization in both hermaphrodite and male germlines (Figs 1C, S1C and S1D). GRAS-1::GFP signal was detected both at germ cell membranes, as confirmed by SYX-4 and Phalloidin staining (S1E Fig) that detects actin filaments, and by tubulin staining (S1F Fig), and near the nuclear envelope in early prophase I, as determined by co-immunolocalization with phosphorylated nuclear envelope protein SUN-1 (Fig 1D; S8-pSUN-1). 44% of S8-pSUN-1 aggregates present in leptotene/zygotene nuclei were in contact with GRAS-1::GFP (n = 215, 13 gonads). S8-pSUN-1 and GRAS-1::GFP signals displayed modest but positive linear correlation at the nuclear envelope of leptotene/zygotene nuclei (S2A Fig; Pearson correlation coefficient 0.177; n = 45, 9 gonads). Moderate correlation is expected given the relative abundance of S8-pSUN-1 and GRAS-1::GFP signals observed. Super-resolution microscopy analysis of a worm line expressing both SUN-1::mCherry and GRAS-1::GFP further supports GRAS-1 localization at the germ cell membrane and in close proximity to the NE, but also revealed foci inside the nuclei suggesting a likely dynamic localization (Fig 1E). Moreover, GRAS-1::GFP localization appears to be largely independent of meiotic DSB production and SC formation, given that GRAS-1 localization is indistinguishable from wild type in *spo-11* and *syp-2* mutants, respectively (S2B Fig). Using a transgenic line expressing GRAS-1-GFP for pull-downs and mass spectrometry analysis, we found proteins previously shown to be expressed in the germline (Fig 1F). GRP-1 appeared as the most enriched protein in all 4 replicates and specific to the GRAS-1::GFP pull-downs. GRP-1 is the worm ortholog of human Cytohesin 1 protein, the main structural and functional partner of CYTIP [24,32], supporting conservation between the proteins. Many of the proteins identified included NE-associated proteins, such as tubulins, PLK-1, importins, the KASH protein KDP-1, and cytoskeleton or spindle structural and motor components. Based on their GO terms or WormBase-described functions and/or localization, germline hits were classified into the following categories: nuclear envelope, spindle/cytoskeleton, meiosis, chromatin, or general germline-expressed proteins. The majority of proteins (667 out of 774, excluding GRAS-1) had a greater than 1.5-fold change suggesting GRAS-1::GFP interactors are localized to/interact with the NE or cytoskeleton (Fig 1G).

### GRAS-1 contributes to normal meiotic progression and accurate chromosome segregation

**Fig 2:**
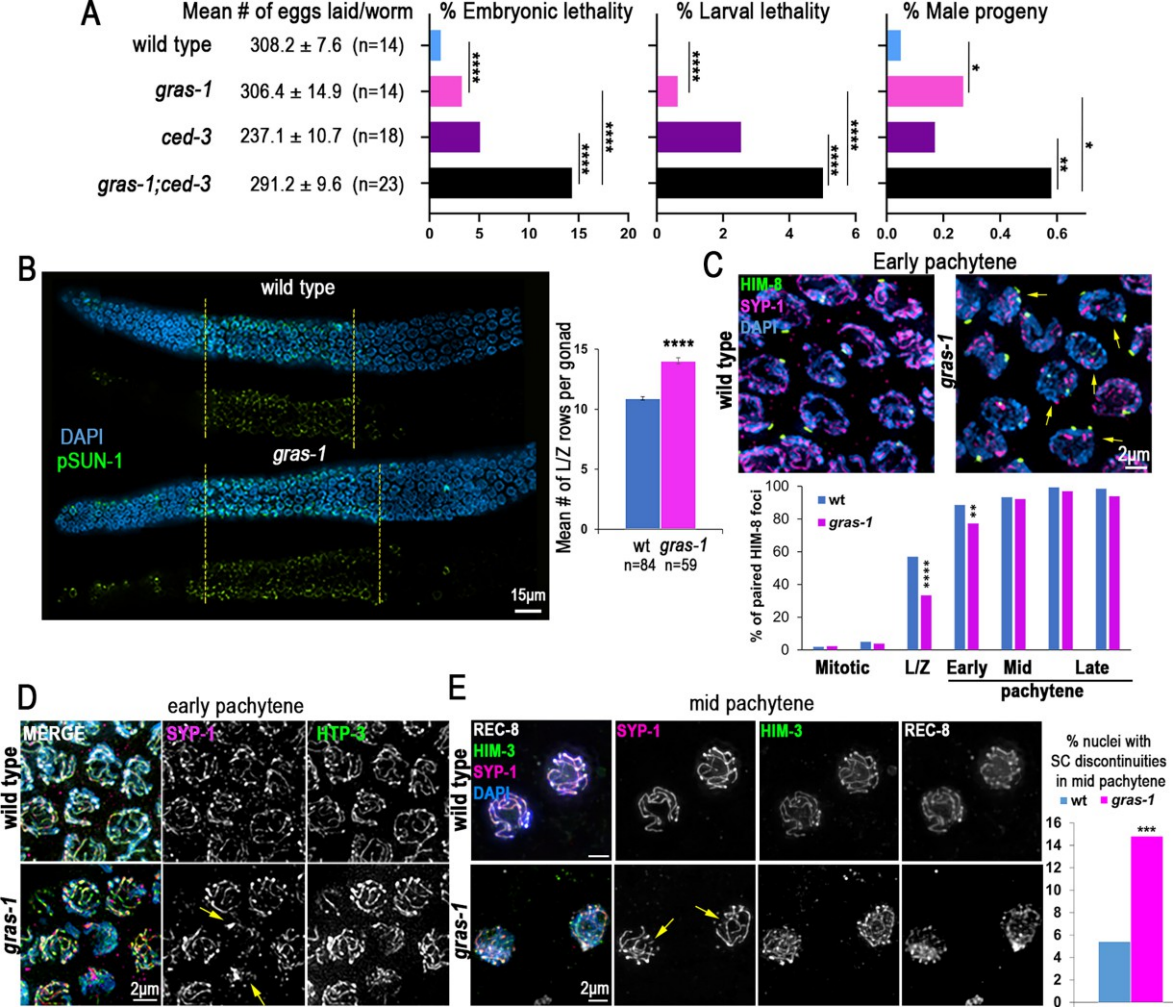
GRAS-1 is required for normal meiotic progression, chromosome pairing, and synapsis. **(A)** Mean number of eggs laid (brood size) ± SEM, the percentage of embryonic lethality, larval lethality, and male progeny are shown for wild type, *gras-1(rj28)*, *ced-3*, and *gras-1(rj28);ced-3*. *p<0.05, **p = 0.0037, ****p<0.0001 by Fisher’s exact test. n = number of P0 worms analyzed from three independent biological replicates. **(B)** Whole mounted gonads of wild type and *gras-1* worms co-stained with anti-S8 pSUN-1 (green) and DAPI (blue). Both merged and S8 pSUN-1 signal only are shown with yellow dotted lines delimiting the region in which complete rows of nuclei presented S8 pSUN-1 signal; n = 30 gonads each. Graph on the right shows the mean number of nuclei in leptotene/zygotene (L/Z) stage per gonad in wild type and *gras-1* worms. ****p<0.0001 by the Mann-Whitney U-test, n = number of worms analyzed from at least 2 independent biological replicates. **(C)** Top, high-resolution images of early pachytene nuclei co-stained with anti-HIM-8 (green), anti-SYP-1 (magenta) and DAPI (blue) from wild type and *gras-1* worms. Yellow arrows indicate nuclei with unpaired HIM-8 signal. Bottom, percentage of nuclei with paired HIM-8 signals (≤0.75μm apart) at different germline stages. ****p<0.0001, **p = 0.008 by the Fisher’s Exact Test; n = 6 gonads each and a minimum of 131 nuclei per zone. **(D)** High-resolution images of wild type and *gras-1* early pachytene nuclei (n = 80 and 50, respectively) from whole mounted gonads co-stained with anti-SYP-1 (magenta), anti-HTP-3 (green) and DAPI (blue). Yellow arrows indicate nuclei with SYP-1 aggregates. **(E)** Left, high-resolution images of lightly squashed gonads of wild type and *gras-1* mid pachytene nuclei co-stained with anti-SYP-1 (magenta), anti-HIM-3 (green), anti-REC-8 (white) and DAPI (blue). Yellow arrows indicate SYP-1 discontinuities. Right, percentage of mid-pachytene nuclei with SC discontinuities in wild type and *gras-1* gonads. ***p = 0.0032, Fisher’s Exact test, n = 253 and 217, respectively, from two biological replicates.

To assess the roles of *gras-1* in the germline, we analyzed the fertility of various *gras-1* alleles including an out-of-frame deletion between the first and second exons (*tm2699*), a partial deletion and frameshift from amino acid 89 (*rj15)*, and whole-gene deletions (*rj27* and *rj28*) (Fig 1A). While all mutants had normal brood sizes, most exhibited a mild but significant increase in the number of eggs laid that failed to hatch (embryonic lethality), elevated levels of male progeny (indicating meiotic chromosome nondisjunction) and increased larval lethality (Figs 2A and S2C). To assess the effects of complete absence of GRAS-1 protein, all subsequent analyses were performed in *gras-1(rj28)* mutants.

Analysis of meiotic progression revealed an extension in the number of rows of nuclei exhibiting phosphorylated SUN-1 (S8 pSUN-1) signal in *gras-1* null mutants compared to wild type (22.8±0.6 and 19.5±0.5, respectively; p = 0.0001, Mann Whitney U-test, Fig 2B). This was accompanied by an increase in the number of rows of nuclei with chromosomes exhibiting the characteristic configuration of leptotene/zygotene stage nuclei in *C*. *elegans* (14±0.3 and 10.9±0.1, respectively; p<0.0001) (Fig 2B). This alteration in meiotic progression is further supported by a delay in Polo-like kinase PLK-2 translocating from the nuclear periphery to synapsed chromosomes by the end of early pachytene (21.9±1 rows of nuclei in *gras-1* and 19±0.5 in wild type, p = 0.0351) (S3A Fig). These data suggest that GRAS-1 contributes to normal meiotic progression.

### GRAS-1 is necessary for timely homologous chromosome pairing and synapsis in an α-importin-independent manner

Delays in meiotic progression during early prophase I can arise from problems in homolog pairing and/or synapsis [11,17,33]. To examine homologous pairing we measured X chromosome pairing throughout meiosis by immunostaining for the X chromosome-specific PC protein HIM-8 [34]. During leptotene/zygotene and early pachytene stages, we observed higher levels of nuclei with two unpaired HIM-8 foci in *gras-1* mutants compared to wild type (p<0.0001 in leptotene/zygotene stage and p = 0.008 in early pachytene, Fisher’s Exact Test) (Fig 2C). However, pairing levels in *gras-1* mutants were indistinguishable from wild type by mid-pachytene and the X chromosomes remained stably paired through the end of pachytene, suggesting that GRAS-1 is only required for achieving timely homologous chromosome pairing in early prophase I.

Early pachytene nuclei with unpaired HIM-8 foci in *gras-1* mutants also showed discontinuous SC or aggregates of the SC central region protein SYP-1, in contrast to the continuous SC tracks detected in wild type (a mean of 2.28±0.24 compared to 0.4±0.08 nuclei with SYP-1 aggregates in *gras-1* and wild type, respectively; p<0.0001, Mann Whitney U-test) (Fig 2D). Discontinuities of the central region of the SC, but not of axial element proteins such as HTP-3, were also detected along chromosomes in mid-pachytene nuclei of whole mounted germlines from *gras-1* mutants compared to wild type (16.9% and 5.1%, respectively; p = 0.002, Fisher’s Exact test) (S3B Fig) and further confirmed on squash preparations (14.8% and 5.4%, respectively; p = 0.0032) (Fig 2E).

The α-importin nuclear transport IMA-2 protein, which was identified in our GRAS-1::GFP pull-downs (Fig 1F), and the Akirin protein AKIR-1 have been proposed to act through parallel pathways to ensure normal chromosome synapsis by promoting import and chromosomal loading of cohesin complex proteins. For instance, *akir-1;ima-2* double mutants exhibit an increased number of nuclei with SC aggregates and discontinuities due to the abnormal loading of axis and cohesin proteins [19]. However, axial element proteins HTP-3 and HIM-3 and the meiosis-specific cohesin REC-8 were correctly loaded on the chromosomes in *gras-1* mutants, suggesting that SC defects may be caused by other mechanisms (Figs 2D, 2E and S3B). Moreover, REC-8 localization was not altered in *gras-1* and *ima-1* or *ima-2*, double and triple mutants (S3C Fig). Interestingly, we detected interaction of GRAS-1 with multiple SC central region proteins, including SYP-3 by western blot analysis of GRAS-1::GFP pull downs (S4A Fig), and SYP-1, SYP-2 and SYP-3 by yeast two-hybrid analysis (S4B Fig). Of note, the interactions detected by yeast two-hybrid analysis between GRAS-1 and either SYP-1 or SYP-2 were weaker than those detected between GRAS-1 and SYP-3. Taken together, these studies support a role for GRAS-1 in promoting timely homologous chromosome pairing and SC assembly in an α-importin-independent manner during early prophase I.

### Early prophase I chromosome movement is limited by GRAS-1 in a dynein-dependent manner

**Fig 3:**
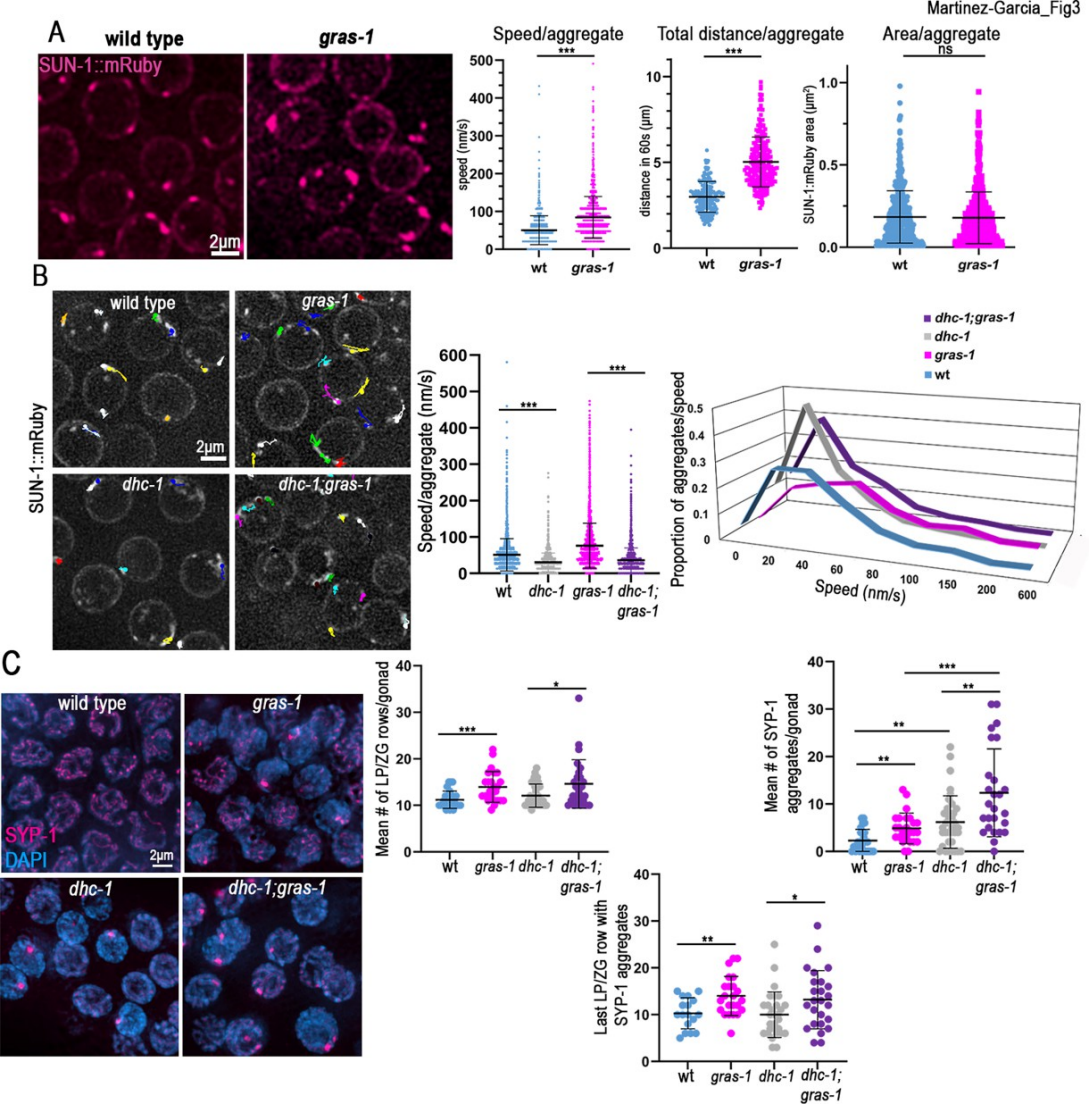
Chromosome movement is limited by GRAS-1 in a dynein-dependent manner during early prophase I. **(A)** Left, snapshots of SUN-1::mRuby live imaging signal (magenta) in wild type and *gras-1* leptotene/zygotene nuclei. Right, dot plots showing the speed (nm/s) of SUN-1::mRuby aggregates, their total distance (μm) traveled in 60s, and their area (μm^2^). ***p<0.001, ns: not significant, by Student’s t-test. n = 173 and 220 aggregates for wild type and *gras-1* for speed and distance and n = 529 and 617 for the area measurement, respectively, from two independent biological repeats. **(B)** Left, snapshots from live imaging of SUN-1::mRuby aggregates showing the paths they travelled in 60s in wild type, *gras-1*, *dhc-1* and *dhc-1;gras-1* leptotene/zygotene nuclei. Right, dot plot displaying the speed (nm/s) of SUN-1::mRuby aggregates and the distribution graph of the aggregates per speed per genotype. ***p<0.0001, Student’s t-test, n = 325, 326, 244, and 377 aggregates, respectively, from between 10 to 13 gonads each, from four independent biological repeats. **(C)** Left, high-resolution images of early pachytene nuclei from wild type, *gras-1*, *dhc-1*, and *dhc-1;gras-1* co-stained with anti-SYP-1 (magenta) and DAPI (blue). Right top, dot plots displaying the number of rows with nuclei at the leptotene/zygotene (LP/ZG) stage per gonad and the mean number of nuclei with SYP-1 aggregates per gonad (n = 26, 25, 36 and 26 gonads, respectively). Right bottom, dot plot showing the last row of nuclei with SYP-1 aggregates per gonad (n = 17, 23, 29 and 25, respectively). *p<0.05, **p<0.01, ***p<0.001 by the Mann-Whitney U-test. Bars show the mean and standard deviation in all graphs.

The delay in homologous pairing and SC assembly observed in *gras-1* mutants is similar to that detected in mutants where chromosome movement is impaired [11,12,35]. Therefore, we assessed chromosome movement by live imaging analysis of SUN-1::mRuby;GFP::H2B aggregates (marking chromosome ends) during meiosis in wild type and *gras-1* young adult worms. Surprisingly, SUN-1 aggregates moved at a greater speed and traveled higher distances in *gras-1* mutants compared to wild type (84.47±1.05 nm/s, average total distance traveled in 60s of 5.03±0.1 μm, and 50.55±0.82 nm/s, average total distance traveled in 60s of 2.99±0.07μm, respectively, p<0.0001 Student’s t-test) (Fig 3A). Importantly, our measurements of SUN-1 aggregates in wild type were comparable to those reported in studies using similar methodology to track chromosome movement [18,36]. To assess a possible role of GRAS-1 in the formation of SUN-1 aggregates, which could affect chromosome movements, we compared the area of SUN-1::mRuby aggregates between *gras-1* and wild type, but we did not observe significant differences (0.179±0.006 μm and 0.184±0.006 μm, respectively, p = 0.5834). Moreover, the increased movement of SUN-1 aggregates is likely not a result of synapsis defects since *syp-1* mutants that lack an SC were shown to have no effect on dynein heavy chain (DHC-1) movement [17].

Since the key motor protein involved in promoting early prophase I chromosome movement in *C*. *elegans* is dynein [35], we examined if the increased SUN-1 speed in *gras-1* mutants was mediated by dynein. Wild type worms partially depleted of *dhc-1* by RNAi (S4C Fig; [36]) exhibited minimal SUN-1 movement with short tracks after 1 minute of imaging and reduced average speed per aggregate (32.66±0.58 nm/s for *dhc-1(RNAi)* and 51.37±0.74 nm/s for wild type, p<0.0001, Student’s t-test) (Fig 3B and S1 Video), comparable with previous reports [36]. The increased speed of SUN-1 observed in *gras-1;EV* (empty vector) worms was lost in *dhc-1(RNAi);gras-1* worms (76.37±1.03 for *gras-1* and 36.21±0.51 for *dhc-1(RNAi);gras-1*, p<0.0001) (Fig 3B, S1 Video). Furthermore, the two types of chromosome movement speeds described for *C*. *elegans* leptotene/zygotene stage nuclei (processive-chromosome motions with higher speeds in one direction and short-distance movements close to one point; [35,36]) observed in wild type and exacerbated in *gras-1* were absent upon *dhc-1* depletion with the majority of aggregates displaying a speed around 20-30nm/s (Fig 3B, rightmost panel). Therefore, the increased speed found in *gras-1* was completely dependent on DHC-1.

Meiotic progression was further impaired in *dhc-1;gras-1* compared to the single *dhc-1* mutant. Compared to wild type, lack of DHC-1 causes a mild extension of the leptotene/zygotene stages (12.08±0.41 and 11.19±0.36 rows of nuclei in *dhc-1* and wild type, respectively, p = 0.18, Mann-Whitney U-test) and a significant increase in the presence of SC aggregates in early pachytene (6.19±0.91 and 2.27±0.46 nuclei with SYP-1 aggregates per gonad in *dhc-1* and wild type, respectively, p = 0.0044) (Fig 3C and [11]). *dhc-1;gras-1* double mutant germlines showed a further increase in the number of nuclei with chromatin exhibiting a leptotene/zygotene stage appearance compared to *dhc-1* alone (14.62±1 leptotene/zygotene rows in *dhc-1;gras-1*, p = 0.0251) (Fig 3C), and significantly higher levels of SYP-1 aggregates compared to single mutants (12.35±1.78 nuclei with SYP-1 aggregates in *dhc-1;gras-1* compared to 6.19±0.91 in *dhc-1* and 4.88±0.63 in *gras-1*, p = 0.0044 and 0.0005, respectively) (Fig 3C). Additionally, more of these persistent leptotene/zygotene-like nuclei with SYP-1 aggregates were detected in later stages of prophase I in *dhc-1;gras-1* than in *dhc-1* mutants (13.2±1.22 rows after leptotene/zygotene entry in *dhc-1;gras-1* compared to 10±0.89 in *dhc-1*, p = 0.0350) (Fig 3C, lower panel). Altogether, these data indicate a DHC-1-dependent role for GRAS-1 in limiting chromosome movement/speed in early prophase. However, exacerbated phenotypes, such as the increased number of SYP-1 aggregates, contrast with the epistatic relationship observed for DHC-1 and GRAS-1 in chromosome movement speed, and suggest that GRAS-1 might exert additional functions in regulating meiotic progression.

### GRAS-1 contributes to normal meiotic DSB repair progression

**Fig 4:**
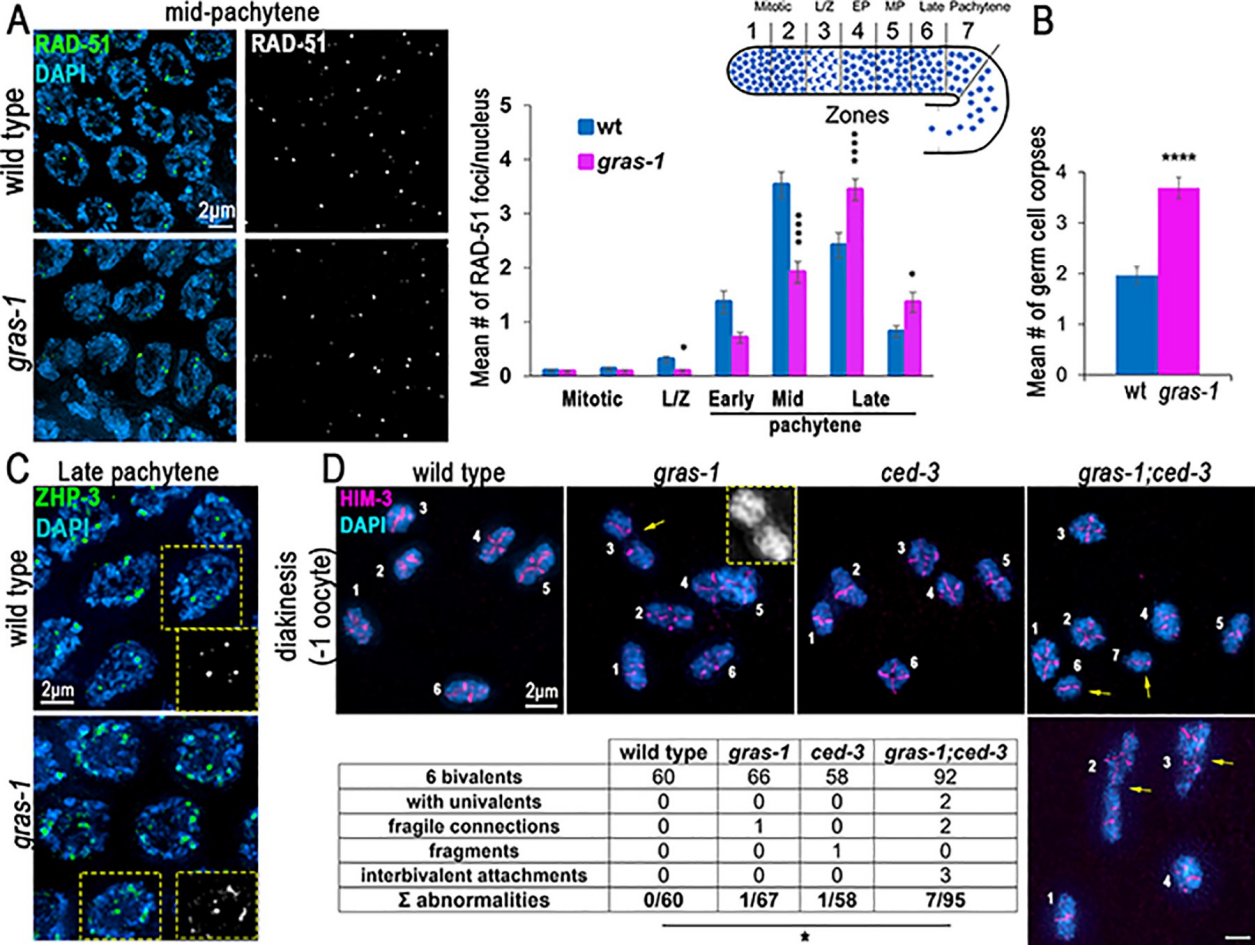
GRAS-1 contributes to normal meiotic DSB repair progression. **(A)** Left, high-resolution images of wild type and *gras-1* mid-pachytene nuclei co-stained with anti-RAD-51 (green) and DAPI (blue). Right, Histogram showing the mean number of RAD-51 foci/nucleus scored along the indicated zones in the germlines of wild type and *gras-1* worms. 3 gonads were scored per genotype and >66 nuclei per zone in two independent biological replicates. Error bars represent the SEM. *p<0.05, ****p<0.0001 by the Mann-Whitney U-test. **(B)** Histogram showing the mean number of germ cell corpses detected in wild type and *gras-1* worms. Error bars represent the SEM. ****p<0.0001, Mann-Whitney U-test, n = 117 and 93 gonads respectively. **(C)** High-resolution images of wild type and *gras-1* late pachytene nuclei stained for ZHP-3 (green) and DAPI (blue). Yellow dotted insets depict ZHP-3 signal in black and white for one of the nuclei in the field showing individual foci in wild type and some ZHP-3 tracks in *gras-1*. 5.99 ± 0.02 and 6.07 ± 0.05 ZHP-3 foci per late pachytene nucleus were detected in wild type and *gras-1*, respectiely; n = 72 and 71 nuclei scored from 15 gonads each. **(D)** Top, High-resolution representative images of diakinesis nuclei stained for HIM-3 (magenta) and with DAPI (blue) from wild type, *gras-1* (yellow arrow indicates a fragile connection), *ced-3*, and *gras-1;ced-3* (yellow arrows indicate univalents in the top and interbivalent attachments in the bottom image). Each individual DAPI-stained body is indicated with a white number. Bottom, table showing the distribution of diakinesis nuclei per genotype that had the normal 6 bivalents or one of the listed abnormalities. *p = 0.0436 between *gras-1;ced-3* and wild type by Fisher’s exact test.

Mutants with altered SC assembly frequently exhibit impaired recombination since the SC is required for normal DSB repair progression and crossover formation [19,21,37,38]. To assess DSB repair in the absence of GRAS-1, we immunostained gonads for RAD-51, a protein involved in strand invasion/exchange steps during homologous recombination [37,39,40]. *gras-1* mutants exhibited a reduction in the number of RAD-51 foci observed per nucleus from leptotene/zygotene through mid-pachytene stages and a slight increase in late pachytene compared to wild type (p = 0.049 for leptotene/zygotene, p<0.0001 for mid-pachytene, and p = 0.039 for late pachytene, Mann-Whitney U-test) (Fig 4A). The increased RAD-51 foci were dependent on the topoisomerase-like SPO-11 protein required for meiotic DSB formation (S4D Fig). Unrepaired recombination intermediates persisting into late pachytene can result in increased germ cell apoptosis [41]. We detected a significant increase in germ cell apoptosis in *gras-1* mutants compared to wild type (3.69±0.21 and 1.97±0.17 mean number of germ cell corpses respectively, p<0.0001, Mann-Whitney U-test) (Fig 4B). Moreover, the increase in germ cell apoptosis was also meiotic DSB-dependent given that apoptosis levels were no longer elevated in *gras-1* mutants in the absence of SPO-11 (S4E Fig). Crossover designation levels were not altered as determined by quantification of the number of foci for ZHP-3, the ortholog of budding yeast Zip3 that marks sites designated for crossover formation in late pachytene nuclei (6.07±0.05 and 5.99±0.02 ZHP-3 foci per nucleus in *gras-1* and wild type, respectively, p = 0.06, Mann-Whitney U-test). However, a delay in the restriction of ZHP-3 signal from tracks to foci was observed in *gras-1* mutants (Fig 4C). Analysis of oocytes at diakinesis revealed 6 bivalents in both wild type and *gras-1* mutants with only one oocyte exhibiting a fragile connection between a pair of homologs in *gras-1* (Fig 4D) [42]. However, analysis of *gras-1* mutants also lacking the *ced-3* caspase [43], which prevents germ cell apoptosis in late pachytene, revealed an increase in the total number of oocytes with chromosome abnormalities (p = 0.0436 compared to wild type, Fisher’s exact test) including univalents, fragile connections, and interbivalent attachments (Fig 4D). This was accompanied by higher levels of embryonic lethality, larval lethality, and male progeny in *gras-1;ced-3* mutants compared to either *ced-3* alone (14.3% vs 5.1% embryonic lethality, p<0.0001; 5% vs 2.5% larval lethality, p<0.0001; and 0.6% vs 0.2% males, p = 0.004, Fisher’s exact test) or *gras-1* alone (3.26% embryonic lethality, p<0.0001, 0.63% larval lethality, p<0.0001, and 0.27% male progeny, p = 0.0314, Fisher’s exact test) (Fig 2A). These combined data suggest that GRAS-1 contributes to normal meiotic DSB repair progression.

### GRAS-1’s function in limiting chromosome movement in early prophase I is regulated by phosphorylation at a C-terminal S/T cluster

**Fig 5:**
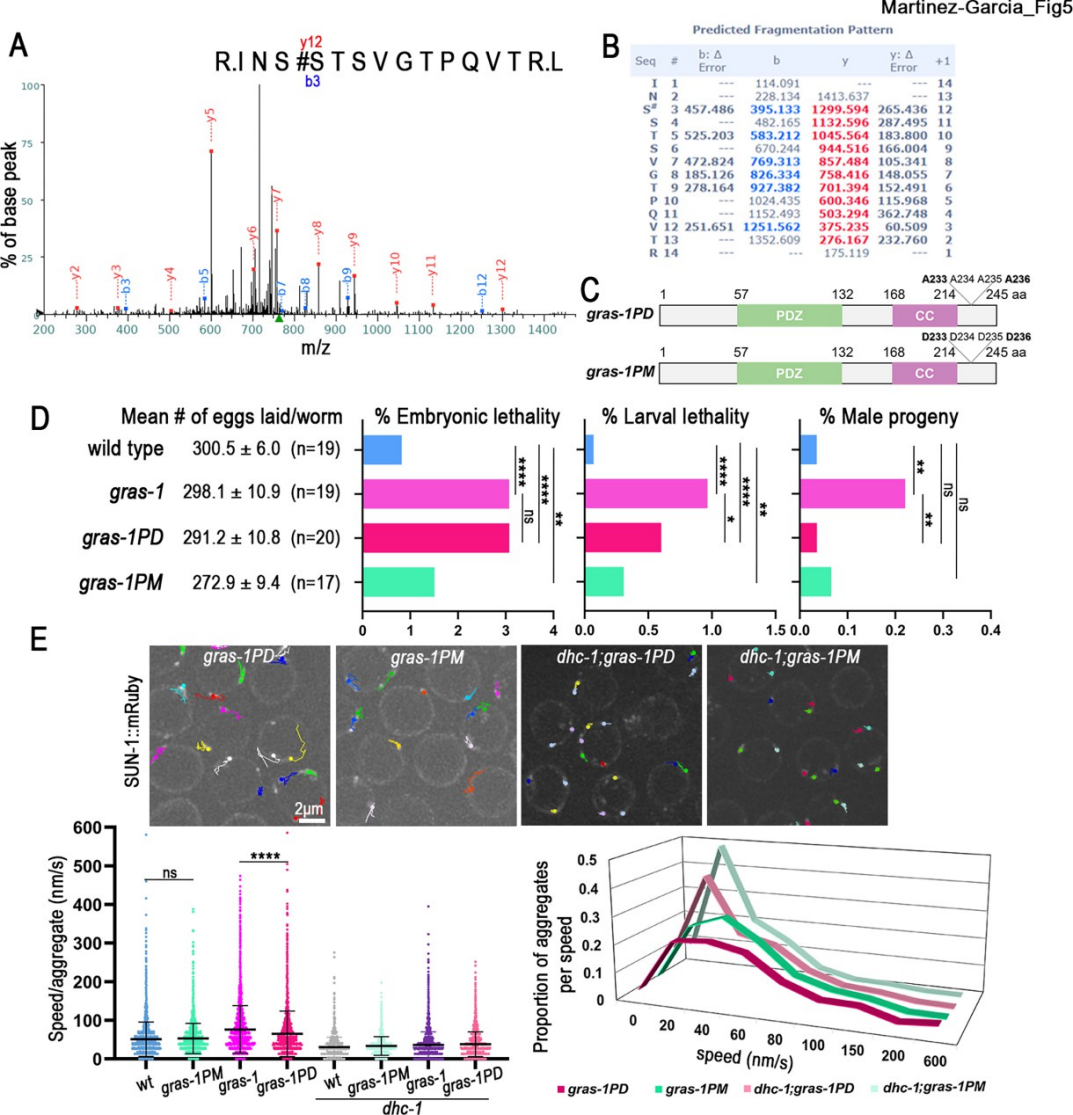
GRAS-1 function in limiting early prophase I chromosome movement is regulated by phosphorylation. **(A)** Mass spectrometry fragmentation spectrum for GRAS-1::GFP peptide INSSTSVGTPQVTRL in the range 200–1400 m/z. The annotated spectrum shows fragment ion species matched between theoretical and measured values. “b-ions” are generated through fragmentation of the N-terminal peptide bond, and “y-ions” through the C-terminal. **(B)** Predicted MS fragmentation pattern and deviations (Δ Error). Analysis of b and y ions is consistent with the phosphorylation of the second serine (S#) in this peptide, corresponding to S233 of the GRAS-1 protein. **(C)** Schematic representation of the proteins encoded by the CRISPR-Cas9 engineered *gras-1PD* (phosphodead) and *gras-1PM* (phosphomimetic) mutants. A: alanine, D: aspartic acid. **(D)** Shown are the mean number of eggs laid (brood size) ± SEM, percentage of embryonic lethality, larval lethality, and males for the indicated genotypes. *p < 0.05, **p <0.01, ****p < 0.0001, ns: not significant, by Fisher’s exact test. n = number of worms for which entire progeny were analyzed. **(E)** Top, snapshot of live imaging of SUN-1::mRuby aggregates and their travelled paths in 60s in *gras-1PD*, *gras-1PM*, *dhc-1;gras-1PD* and *dhc-1;gras-1PM* leptotene/zygotene nuclei. Bottom, dot plot displaying the speed (nm/s) of SUN-1::mRuby aggregates and the distribution graph of the aggregates per speed for the indicated genotypes. Worms were grown in bacteria containing the empty-vector or *dhc-1(RNAi)* construct. ****p<0.0001, ns: not significant by Student’s t-test, n = 325, 343, 326, 245, 244, 195, 377 and 247 aggregates per genotype as shown in figure, from 9 to 13 gonads and at least two independent biological repeats. Error bars represent the mean ± SD.

Analysis with different protein phosphorylation prediction programs (Kinase 2.0, NetPhos 3.1 and PHOSIDA) identified S233 and S236 as putative phosphorylation sites within a S/T cluster domain (SSTS) at the C-terminus of GRAS-1 (Fig 1A). These sites are conserved in human CYTIP (S269 and S270) and GRASP/Tamalin (S293), the former being strongly conserved in other vertebrates (PER viewer) [44]. *In vivo* phosphorylation of GRAS-1 at this S/T cluster was confirmed by mass spectrometry analysis (Fig 5A and 5B; shown is phosphorylation at S233). Since more than one residue at the SSTS cluster may be phosphorylated, we used CRISPR-Cas9 to edit all four amino acids to either alanine (A) or aspartic acid (D) to generate phosphodead (*gras-1PD*) and phosphomimetic (*gras-1PM*) mutants, respectively (Fig 5C). Analysis of *gras-1PD* and *gras-1PM* mutants revealed a normal number of eggs laid but increased embryonic and larval lethality compared to wild type (Fig 5D). *gras-1PD* mutants exhibited embryonic and larval lethality levels similar to *gras-1* null (3.1% embryonic lethality in both, p>0.99, and 0.6% larval lethality in *gras-1PD* and 0.97% in *gras-1*, p = 0.031, Fisher’s exact test). In contrast, the number of male progeny in phosphodead or phosphomimetic mutants was indistinguishable from wild type (Fig 5D, right panel), suggesting that X chromosome segregation is regulated independently from phosphorylation of GRAS-1. To assess whether GRAS-1 phosphorylation is required for its role in limiting chromosome movement in early prophase I, we analyzed the speed of SUN-1::mRuby aggregates in *gras-1PD* and *gras-1PM* mutants (Fig 5E and S2 Video). The inactivation of the phosphorylation domain in *gras-1PD* produced a higher average speed per aggregate compared to wild type (65.18±1.12 nm/s and 51.37±0.74 nm/s, respectively, 325 and 245 aggregates, p <0.0001, Student’s t-test), but not as elevated as in the *gras-1* null mutant (73.17±1.09 nm/s, 270 aggregates, p<0.0001). In contrast, mimicking a phosphorylated SSTS domain resulted in chromosome movement speeds similar to those observed in wild type (53.05±0.65 nm/s in *gras-1PM*, 343 aggregates, p = 0.0872). Depletion of dynein by RNAi in the phosphodead and phosphomimetic mutants resulted in SUN-1 aggregates indicating non-processive chromosome motions with slower speeds (Fig 5E, lower right panel). These results suggest that GRAS-1 function in limiting chromosome movement is regulated by phosphorylation at these conserved C-terminal residues.

### GRAS-1 shares partial functional conservation with human CYTIP

**Fig 6:**
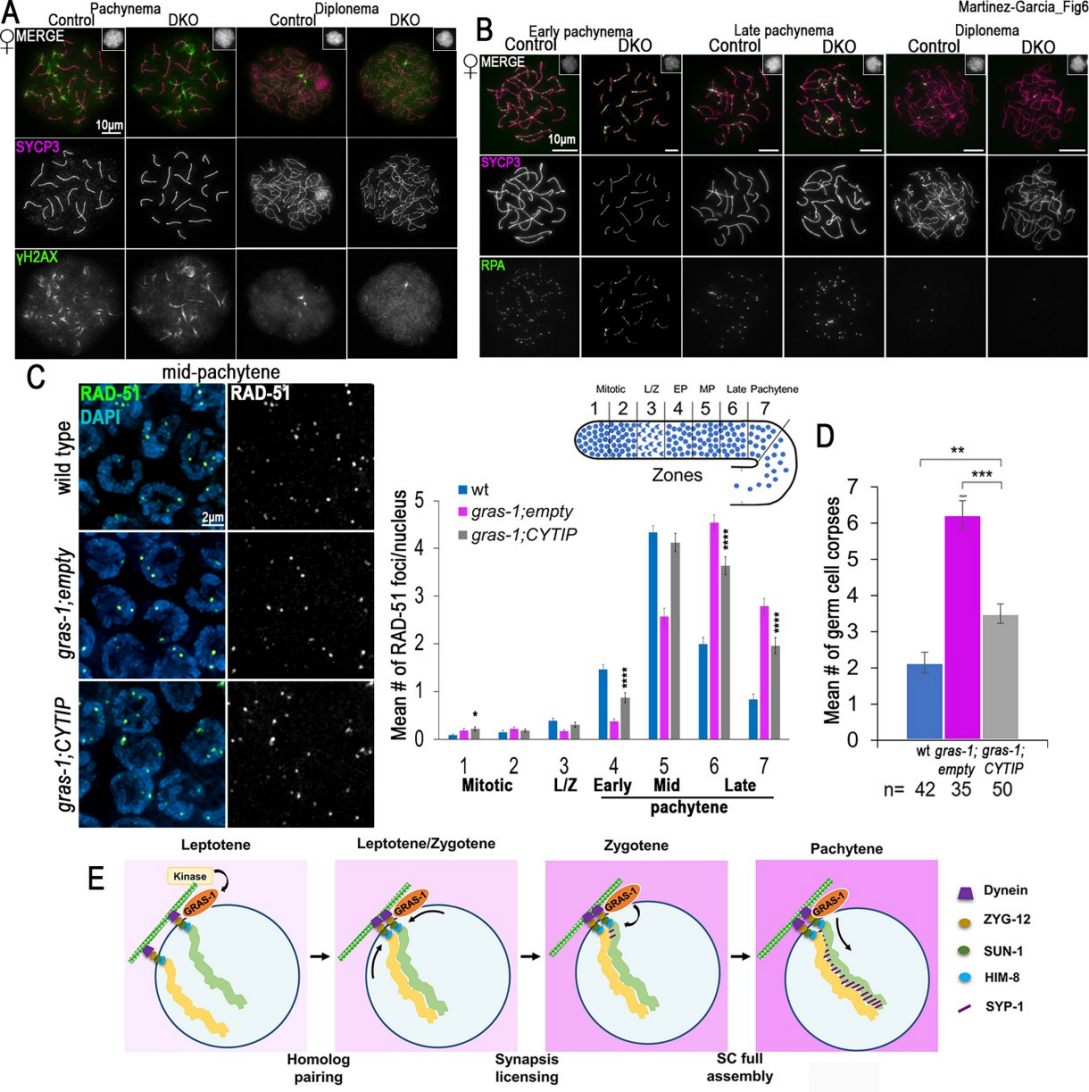
GRAS-1 shares partial functional conservation with human CYTIP. **(A)** Chromatin spreads from mid meiotic prophase (pachynema) and late meiotic prophase (diplonema) control and *Tamalin-Cytip* DKO *Mus musculus* oocytes co-immunostained with anti-SYCP3 (magenta) and anti-γ-H2AX (green). Insets show normal chromatin morphology (DAPI). n = 50 cells per mouse and 3 mice per genotype. **(B)** Chromatin spreads from early and late pachynema and diplonema, control and *Tamalin-Cytip* DKO *Mus musculus* oocytes co-immunostained with antibodies against SYCP3 (magenta) and RPA (green). Insets show normal chromatin morphology (DAPI). **(C)** Left, high-resolution images of mid-pachytene nuclei from wild type, *gras-1;empty* and *gras-1;CYTIP* germlines stained with anti-RAD-51 (green) and DAPI (blue). Right, Histogram showing the mean number of RAD-51 foci/nucleus scored along the germlines for the indicated genotypes. 5–6 gonads were scored per genotype in two independent biological replicates. Error bars represent the SEM. *p<0.05, ****p<0.0001 by the Mann-Whitney U-test. **(D)** Histogram showing the mean number of germ cell corpses of wild type (*gras-1;empty* and *gras-1;CYTIP* worms. Error bars represent the SEM. **p<0.01, ***p<0.001, Mann-Whitney U-test, n = 42, 35 and 50 gonads, respectively. **(E)** A model for the role of GRAS-1 during *C*. *elegans* meiosis. We propose that GRAS-1 bridges the cytoskeleton, the LINC complexes, and chromosomes to limit chromosome movement in a phosphorylation-dependent manner and license synapsis during early prophase I. A single pair of homologous chromosomes (yellow and green) is shown for simplicity within a nucleus delimited by the nuclear envelope (dark blue line) and attached to the LINC complex and a single microtubule (green/white checkered bar).

The fact that GRAS-1 protein structure, phosphorylation sites, and reproductive tissue expression are conserved in mammals (Figs 1A and S1A) suggests that similar functions could be performed by either CYTIP and/or GRASP/Tamalin. To test this possibility, we first examined *Tamalin*, *Cytip* DKO mouse mutants (S5A Fig). The mouse mutants had fertility rates, testis weight, and seminiferous tubule morphology equivalent to littermate controls (S5B Fig). Analysis of meiotic progression in chromatin spreads from both male and female *Tamalin*, *Cytip* DKOs immunostained with γH2AX to assess DSB formation and SYCP3 to examine chromosome synapsis did not reveal any defects compared to controls in oocytes (Fig 6A) and spermatocytes (S5C Fig). Analysis of DSB repair progression by immunostaining meiotic prophase I cells for RPA revealed normal levels relative to controls in oocytes (Fig 6B), and the ATR DNA damage response kinase and RPA in spermatocytes (S5D and S5E Fig). The formation of the central element of the SC also showed normal progression in oocytes (S6A Fig). Finally, the number of crossover recombination events determined by assessing MLH1 foci in spermatocytes and CDK2 in oocytes from *Tamalin*, *Cytip* DKO mice was the same as in wild type (S6B Fig, 1.08 ± 0.02, n = 416, and 1.09 ± 0.02, n = 409, MLH1 foci on chromosomes in the DKO and the control, respectively, p = 0.7641 Mann-Whitney test; S6C Fig, 3.18 ± 0.02, n = 451, and 3.14 ± 0.03, n = 427, CDK2 foci in the DKO and the control, respectively, p = 0.2332). These results are similar to the crossover analysis in the worm *gras-1* mutant in which levels of ZHP-3 foci were indistinguishable from wild type.

Even though mutant *Tamalin*, *Cytip* DKO mice did not present obvious fertility or meiotic defects, we cannot exclude the possibility of additional redundancy. Moreover, GRAS-1 function could still be conserved to a lesser extent or diverged in mice but not in other vertebrates. To assess functional conservation with the human orthologs, we complemented *gras-1* null worms with the human cDNA of CYTIP, the ortholog with the highest sequence similarity. Using the SKI LODGE system [45] we introduced a cassette into chromosome III with expression of the human coding sequence driven by the *pie-1* germline-specific promoter (S6D–S6F Fig). We examined DSB repair progression by RAD-51 immunostaining of germlines from wild type, *gras-1* null carrying an empty vector cassette inserted in chromosome III, and *gras-1* expressing human CYTIP (Fig 6C). *gras-1;CYTIP* exhibited a partial rescue relative to *gras-1* with RAD-51 levels increasing in early pachytene, albeit not reaching the same levels as in wild type until mid-pachytene (0.87±0.10 in *gras-1;CYTIP* and 1.47±0.10 foci/nucleus in wild type, p<0.0001, Mann-Whitney U-test), and a partial reduction in the levels observed in late pachytene (3.64±0.19 and 1.96±0.17 foci in late pachytene in zones 6 and 7, respectively, in *gras-1;CYTIP*; 1.99±0.14 and 0.84±0.11 in wild type, p<0.0001). This partial rescue might be due to lower levels of HsCYTIP expression compared to GRAS-1 and/or a requirement for co-expression of Tamalin for full function. Therefore, human CYTIP expression in *gras-1* null mutants resulted in an intermediate phenotype between *gras-1* null and wild type. Similarly, we observed reduced levels of germ cell corpses in *gras-1* worms complemented with CYTIP compared to *gras-1* null (6.23±0.40 in *gras-1;empty* and 3.5±0.27 in *gras-1;CYTIP*, p<0.0001, Fisher’s exact test), but not a complete reversion to wild type levels (2.14±0.28, p = 0.001) (Fig 6D). A partial rescue of the SC assembly defects was also observed with lower levels of SC aggregates in early pachytene (S6G Fig). These results suggest that GRAS-1 protein function could play similar roles in vertebrates, but the divergence of the proteins, the duplication, and potential additional redundancy might affect the processes involved.

## Discussion

Early prophase I events are key determinants of correct chromosome segregation at meiosis I and therefore need to be tightly coordinated. In the present study, we uncover a layer of regulation for these events mediated by the conserved GRAS-1 protein. We propose that GRAS-1 connects the stabilization of homologous chromosome pairing with the licensing of SC formation between homologs by limiting chromosome movement during early prophase I (Fig 6E).

GRAS-1 expression increases in germline nuclei during the transition from mitosis into meiotic prophase I (Figs 1C and S1B) and GRAS-1 localizes near the germ cell NE (Figs 1E, S1E and S1F). GRAS-1 localization seems to be more dispersed compared to the membrane actin fibers and cell membrane components such as SYX-4 [46] (S1E Fig), and while it contacts the NE cytologically it is also detected as foci inside the nucleus (Fig 1D and 1E). The apparent dynamic localization of GRAS-1 may help in coordinating functions at different locations within the cell such as the membrane, the nuclear periphery, and occasionally inside the nucleus. Interactions with SYP-1, SYP-2 and SYP-3 detected by yeast two-hybrid analysis, and GRAS-1::GFP immunoprecipitation of SYP-3, support a direct interaction with nuclear proteins of the synaptonemal complex (S4A and S4B Fig). However, we cannot exclude the possibility that they might occur indirectly via the LINC complex at the NE. Furthermore, mass spectrometry results from GRAS-1::GFP pull-downs show that GRAS-1 may interact directly or indirectly with numerous cytoskeleton proteins such as tubulin and actin subunits, which is further supported by its similar localization to that of actin filaments as shown by Phalloidin staining (Figs 1F, 1G and S1E). The pull-downs also identified various proteins with functions in chromatin and chromosome segregation such as separase, a caspase-related protease that regulates sister chromatid separation [47,48]; the protein phosphatase PP1 orthologs GSP-1/2 with various roles including regulation of sister chromatid cohesion upon entrance into meiosis [49,50]; and PLK-1, whose meiotic role in phosphorylating key chromosome movement regulators and SC components could be an important effector for GRAS-1 function during early prophase I [12,16,51]. Several proteins with functions related to the NE, spindle, and chromosome movement were also found as GRAS-1 putative interaction partners. LMN-1, which plays an important role in chromosome movement, supports a connection for GRAS-1 with structural components of the NE [18,34]. The motor protein kinesin KLP-17 provides an additional target by which GRAS-1 chromosome movement functions could be connected. Kinesins produce opposite movements of cargo proteins to dyneins and the *C*. *elegans*-specific KLP-17 protein is expressed in the germline, has microtubule binding activity, and has been proposed to have chromosome movement and segregation activity [52–54]. The KASH domain protein KDP-1 is implicated in cell cycle progression and its localization depends on SUN-1 in the germline [55]. The LINC complex protein KDP-1 interacts with SUN-1 or UNC-84 at the NE and could interact with other SUN-1 partners, although its role during meiosis requires further investigation. Finally, nucleoporins and importins identified in the pull-downs (NPP-3, NPP-13, IMA-2, and IMA-3) have been implicated in regulating chromosome attachment to the NE, chromosome movement, meiotic recombination, chromosome segregation, and the timely incorporation of SC proteins [19,56]. However, our analysis of *gras-1* in combination with *ima-1* and *ima-2* mutants does not support them acting in the same pathway.

Our data indicate that GRAS-1 acts to limit chromosome movement (Fig 3). GRAS-1 could impose resistance to the free movement of chromosomes from outside of the nucleus when they find a homologous partner, thereby stabilizing that connection (Fig 6E). In *C*. *elegans*, similarly to mice and *S*. *pombe*, the cytoskeletal forces driving the movement of chromosomes from outside the nucleus are controlled by microtubules and the motor protein dynein connecting to the chromosome-LINC complex [4,11,35]. GRAS-1 may function in the same pathway and limit the action of dynein and microtubules since dynein depletion in the absence of GRAS-1 results in chromosome movements similar to those in the dynein mutant alone (Fig 3B). Although mutations in co-chaperone FKB-6 also increase meiotic chromosome movement [17], that study tracked movement by following DHC-1 instead of SUN-1 aggregates and physically immobilizing worms rather than using a chemical paralyzing agent, therefore limiting comparisons based on these differences. Nevertheless, GRAS-1 must act through a different pathway since *fkb-6* mutants showed decreased resting time between chromosome movements, whereas aggregates in *gras-1* had increased general speeds (Fig 3A). Further, the *fkb-6* mutant in combination with either *dhc-1* depletion or a *zyg-12* mutant did not exhibit exacerbated defects in SC formation or chromosome pairing, in contrast with *dhc-1(RNAi);gras-1* mutants where these defects are accentuated (Fig 3C). Moreover, FKB-6 was not identified in GRAS-1::GFP pull-downs, and its localization was more dispersed throughout the cytoplasm in contrast to the membrane localization for GRAS-1. Further, FKB-6 expression was not meiosis-specific, which is connected with a role for FKB-6 in regulating microtubule formation and mitotic segregation in the *C*. *elegans* germline [17]. Additionally, cytoplasmatic protein vinculin/DEB-1 has also been proposed to limit the movement of LINC complexes and produce abnormal synapsis [57]. We believe GRAS-1 functions in a different way than vinculin/DEB-1 because of the differences in localization and phenotypes: *deb-1* mutants had a high number of univalents at diakinesis, defects in the loading of proteins along meiotic chromosome axes (which could be the cause of the severe synapsis defects observed), and their pairing defects are opposite to that in *gras-1* since they initially have the same level of pairing as wild type worms, but then homologs do not achieve complete pairing in most pachytene nuclei.

The excess chromosome movement found in the absence of GRAS-1 could be the reason for the extension in leptotene/zygotene stages, the pairing delays, and the altered DSB repair progression observed in the germline (Figs 2B, 2C and 4). In addition, the relation of GRAS-1 with the SC might be a consequence of its role in chromosome movement and its interactions with the LINC complex-motor protein system. The SC assembly problems and discontinuities observed in the *gras-1* mutant could arise from the unstable chromosome movements. However, GRAS-1 could be involved in transmitting additional signals once homologs find a partner, since some of the GRAS-1::GFP signal is detected inside the nucleus, it interacts with SYP-3, and in *dhc-1;gras-1* double mutants there were more instances of SC aggregates and those appear in late pachytene (Figs 3C and 6E). One possibility is that GRAS-1 helps license the initial assembly of the SC from the PC ends of paired chromosomes, so that in the absence of GRAS-1 homologs do not stay together long enough and the imported SC proteins self-aggregate. However, if that were the case we would expect the SC defects to affect most nuclei, as observed for the defects in chromosome movement. Alternatively, GRAS-1 may regulate the loading of SC proteins via a yet unknown mechanism, given the incomplete polymerization of SYP-1 observed in *gras-1* mutants at mid-pachytene stage (Fig 2D and 2E). This is further supported by the presence of SYP-3 in GRAS-1::GFP pull-downs assessed on westerns and interactions with SYP-1/2/3 in yeast two-hybrid assays (S4A and S4B Fig). GRAS-1 has PDZ and coiled-coil domains, usually involved in protein-protein interactions, and both seem to be needed for interactions with the SC proteins given that these are either lost or weakened with truncations in either the PDZ or the coiled-coil domain respectively in the ΔN and ΔC GRAS-1 constructs by yeast two-hybrid analysis (S4B Fig). However, the part of the PDZ domain still remaining in the *gras-1(rj15)* allele appears to be sufficient for partial function, since that mutant did not exhibit embryonic lethality or increased levels of male progeny along with only weak larval lethality compared to the null mutants (S2C Fig). If GRAS-1 regulates SC assembly and/or loading, it does so in a manner that is distinct from the combined role of Akirin with importins [19] since we did not find evidence of cohesin or axial element defects in *gras-1* mutants alone, or in combination with *ima-1* and *ima-2* (Figs 2D, 2E and S3B–S3C).

GRAS-1 is conserved in animals, and the gene underwent a duplication event in chordates resulting in CYTIP and GRASP/Tamalin (Fig 1A and 1B). All three proteins carry PDZ and coiled-coil domains. In addition, they carry a disorganized C-terminal region (longer in the mammalian orthologs) that could be involved in regulating their function since a phosphorylated serine in the S/T cluster is conserved in both human CYTIP and GRASP. In addition, GRAS-1 protein interactions might also be conserved since mammalian CYTIP and GRASP have been found to interact with Cytohesin-1 [22,24,32] and the worm ortholog, GRP-1, was a top and specific hit in all GRAS-1::GFP pull-down experiments. Similar to worm *gras-1* mutants, *Tamalin*, *Cytip* DKO mice did not exhibit severe fertility defects or crossover recombination problems (Fig 6). However, DKO mice also did not show defects in meiotic progression, chromosome synapsis, and DSB repair progression (Figs 6, S5 and S6). Meiotic progression defects may be more easily detected in *gras-1* mutant worms because of the spatiotemporal organization of meiosis within intact worm gonads that facilitates the observation and quantification of subtle defects compared to individual cell spreading techniques in mouse samples. Therefore, there may be altered chromosome movements in the DKO mice, similar to *gras-1* mutant worms, that we could not measure. Gene duplication divergence might also explain these differences because CYTIP and GRASP are sometimes expressed in different tissues and often have distinct roles [26,58,59]. However, protein structure and functions could still be conserved throughout evolution to partially complement worm GRAS-1 function with the closest human ortholog, CYTIP (Figs 6 and S6D–S6G). Moreover, there could be differences between the mouse and human proteins, or subtle phenotypes or timing issues in the double KO that we could not detect.

In conclusion, we propose a model for the conserved GRAS-1 protein during meiosis in which its localization and protein interactions limit the movement of chromosomes in early prophase I. GRAS-1 may function to stabilize connections between homologs by connecting the NE environment with cytoskeletal forces to license SC assembly (Fig 6E).

## Materials and methods

### Worm strains and growth conditions

N2 Bristol worms were used as the wild type background. Lines were cultured under standard conditions as in [60]. Some mutant lines were obtained from the Caenorhabditis Genetics Center (CGC) and from the National BioResource Project for the nematode *C*. *elegans* (NBRP, Japan). *gras-1* mutant lines were generated using the CRISPR-Cas9 system [61,62]. A deletion from +295 to 189 post termination codon nucleotides was initially generated (*rj15*). Full deletion lines from -28 to 52 post termination codon nucleotides (*rj27*) and start codon to 27 nucleotides after the stop codon (*rj28*), not affecting the promoter of operon CEOP1424, were generated using sgRNA GTTTATCTCTGAACACTCAT and the PAM sequence was mutated from GGG to AGA. The *gras-1(rj28)* allele was used for these studies since the deletion in *rj27* partly extends into the promoter.

A *gras-1*::*gfp* line was generated using sgRNA TACTAGAGACGCGTGACTTG, a linker (ggcggcagcggc) and GFP sequence (pPD95.67) before the stop codon. The guideRNA sequence was mutated to avoid re-cutting. Phosphodead (*gras-1PD*) and phosphomimetic (*gras-1PM*) mutants were produced using the sgRNAs CACGCTTTACGAACTTGAT and TTTACTAGAGACGCGTGACT, respectively, and by changing the PAMs or sgRNA region to synonymous codons to avoid re-cutting. Changes in codons 233 to 236 were made so that SSTS amino acids were mutated into AAAA or DDDD, respectively. All three CRISPR-Cas9-engineered lines were produced by SunyBiotech (Fu Jian, China).

A complementation line expressing HsCYTIP (Dharmacon, MHS6278-202807568) cDNA was generated using the SKI-LODGE system [45]. HsCYTIP was inserted into *wbmls60[pie-1p*::*3xFLAG*::*dpy-10 crRNA*::*unc-54 3’UTR*, *III]* using *dpy-10* crRNA as a target and a PCR template with homology arms including a GFP artificial intron (pPD95.67, gtaagtttaaacatatatatactaactaaccctgattatttaaattttcag) before the CYTIP start codon. CRISPR-generated mutations were Sanger-sequenced (Macrogen). CGC and NBRP mutants were outcrossed at least 6 times with N2. CRISPR-Cas9 generated lines were outcrossed at least 4 times with N2. A full list of the strains used in this study can be found in S1 Table.

### Yeast two-hybrid analysis

GRAS-1 was found in a yeast two-hybrid screen designed to identify proteins interacting with the SC central region protein SYP-3 [21]. This was confirmed by using GRAS-1 full length, ΔNt^69-245^, and ΔCt^1-163^ cloned into Gateway destination vector pVV213 (activation domain, AD). pVV212 (Gal4 DNA binding domain, DB) was used to clone SYP-1/2/3/4 full length, N-terminal, and C-terminal constructs described in [21,63]. Strains were mated on YPD and selected on SC Leu- Trp- plates as described in [64,65].

### Immunoprecipitation and MS analysis

24h post-L4 worms expressing GRAS-1::GFP were collected, frozen, and homogenized, and an anti-GFP antibody used for immunoprecipitation as in [66,67], in four independent experiments. To identify the interacting proteins in GRAS-1::GFP pull-downs and examine the phosphorylation status of GRAS-1, a proteoExtract protein precipitation kit (Calbiochem, #539180) was used followed by mass spectrometry analysis (Taplin Biological Mass Spectrometry Facility, HMS, MA). Protein interactors were curated using 4 independent controls (wild-type worms, a *pie-1*::GFP line generated for the complementation analysis, and two unrelated GFP-tagged lines) and the 4 independent GRAS-1::GFP experiments using the Normalized Spectral Abundance Factor method [68], normalizing by protein weight, the total number of peptides per experiment, substituting the hits not found in a particular experiment by the 100 times lowest percentile, and bait correction factor. Fold-change relative to the controls was calculated using the average of the 4 experiments normalized to the bait peptides. T-student test was used to determine which proteins with greater than 1.5-fold change were statistically significant and corrected by the number of hypotheses. A volcano plot was generated using the log2 of the fold-change and -log10 of the p-value.

### *C*. *elegans* immunofluorescence and imaging methods

*C*. *elegans* gonads from 24 hour post-L4 hermaphrodites were dissected and whole-mounted on slides as in [37] using 1% paraformaldehyde fixation, or 4% for the α-RAD-51 time course analysis. A list of primary antibodies used in this study along with their corresponding dilutions can be found in S2 Table. Secondary antibodies were purchased from Jackson ImmunoResearch Laboratories (West Grove, PA) as AffiniPure IgG (H+L) with minimum crossreactivity: α-rabbit Cy3, α-goat Cy3 (1:200); α-chicken Alexa 488, α-rabbit Alexa 488, α-guinea pig Alexa 488 (1:500); and α-rabbit Cy5, α-goat Cy5, α-guinea pig Cy5, α-chicken Alexa 647 (1:100).

High-resolution imaging was performed with a IX-70 microscope (Olympus, MA) at 0.2μm Z-intervals usually dividing the gonad in 7 equally sized zones from the distal tip with a cooled CCD camera (CH350; Roper Scientific, AZ) driven by the DeltaVision Imaging System (Applied Precision, GE Healthcare). Fixed samples were imaged using a 100x objective (N.A. 1.4), 10X eyepieces, and an auxiliary magnification lens of 1.5X for imaging diakinesis oocytes. Images were deconvolved using a conservative ratio and 15x cycles with SoftWorx 3.3.6 software from Applied Precision, and processed with Fiji ImageJ [69].

Super-resolution imaging of 24 hour post-L4 *gras-1*::*gfp;sun-1*::*mRuby* worms was performed with an OMX 3D-Structured Illumination microscope with focus drift collection after point-spread function assessment (Nikon Imaging Center, Harvard Medical School).

### Quantification of S8 pSUN-1 and GRAS-1::GFP contact

The total number of S8 pSUN-1 aggregates present in leptotene/zygotene nuclei exhibiting representative GRAS-1::GFP signal were quantified from high-resolution Z-stacks. The fraction of S8 pSUN-1 aggregates in contact with GRAS-1::GFP signal was then quantified, with contact being defined as immediately adjacent or overlapping positive signal in both the S8 pSUN-1 and GRAS-1::GFP channels.

### Colocalization of S8 pSUN-1 and GRAS-1::GFP

Leptotene/zygotene nuclei staining positively for S8 pSUN-1 were randomly selected from high-resolution Z-stacks. The region of the inner nuclear envelope where chromosomes cluster during pairing (i.e., the region corresponding to adjacent or overlapping DAPI and NPC staining) was selected as a region of interest (ROI). A line-plot of raw pixel intensities for S8 pSUN-1 and GRAS-1::GFP signals along the ROI was generated using the Multichannel Plot Profile plugin for Fiji ImageJ [70]. Pixel intensity values for each ROI were used to calculate the Pearson correlation coefficient as a measure of normalized covariance of the signals. Coefficients calculated from 45 leptotene/zygotene nuclei across 9 gonads were then averaged.

### Pairing measurements

Quantitative time course analysis of homologous chromosome pairing was assessed by immunostaining 24 hour post-L4 dissected gonads with α-HIM-8. Gonads were divided into seven 512x512 pixel zones (pixel size = 0.0663 microns at 100x), with Zone 1 starting approximately three nuclear diameters away from the distal gonad tip as in [71]. HIM-8 foci were considered paired if ≤0.75μm apart (distances measured in 3D). Two independent biological replicates and a total of 6 gonads were scored for each genotype. The average number of nuclei scored per zone in wild type and *gras-1* was respectively: zone 1 (n = 160, 134), zone 2 (n = 142, 133), zone 3 (n = 144, 150), zone 4 (n = 157, 167), zone 5 (n = 165, 178), zone 6 (n = 136, 165), and zone 7 (n = 131, 145).

### RAD-51 and ZHP-3 time course analyses

Whole-mounted gonads from 24 hour post-L4 hermaphrodites immunostained either for RAD-51 or ZHP-3 were divided into seven equal-size zones with two independent biological replicates per comparison. Fiji plugin Cell Counter (https://imagej.nih.gov/ij/plugins/cell-counter.html) was used to track in 3D the number of foci for each nucleus in a zone. The average number of nuclei scored per zone for the RAD-51 analysis was: zone 1 (n = 97.8), zone 2 (n = 114), zone 3 (n = 115), zone 4 (115.6), zone 5 (n = 113.4), zone 6 (n = 108), and zone 7 (n = 101.6). The number of nuclei scored in zones where ZHP-3 signal was restricted to individual foci was 72 (wild type) and 71 (*gras-1*).

### Plate phenotyping

Between 10 to 15 L4-stage hermaphrodites for each genotype were placed on individual NGM plates freshly seeded with *E*. *coli* OP50 to score the total numbers of eggs laid (brood size), embryonic lethality (number of unhatched eggs/total number of eggs laid), larval lethality (number of dead larvae/total number of hatched eggs) and male frequency (number of males /total number of adult worms). Individual P0 worms were moved every 24 hours to new plates for four consecutive days to score entire brood sizes.

### RNAi by feeding

Feeding RNA interference experiments were performed as in [72] using HT115 bacteria expressing pL4440 empty vector as a control and bacteria expressing dsRNA for the gene of interest from the Ahringer RNAi library (*ima-2* F26B1.3, *dhc-1* T21E12.4) (Source Bioscience). Between three to five L4-stage worms were placed per plate (in a minimum of 2 plates per genotype per replicate) and grown at room temperature. F1 L4 animals were transferred to newly seeded RNAi plates and 24h post-L4 worms were analyzed. Alternatively, P0 L1-stage animals were placed in RNAi plates at 25°C and analyzed 24h post-L4 stage when performing *dhc-1* depletion experiments.

### Germ cell apoptosis experiments

The number of germ cell corpses per gonad arm was scored in 20h post-L4 stage worms as in [73]. A minimum of 30 gonads were scored for each genotype using a Leica DM5000B fluorescence microscope.

### Bioinformatics and databases

The evolutionary tree of GRAS-1 family protein members was obtained from TreeFam (2019 TF316315, [74], http://www.treefam.org/). Degree of conservation between CeGRAS-1 and HsCYTIP or HsGRASP was calculated using NCBI-blast (https://blast.ncbi.nlm.nih.gov/Blast.cgi) and NCBI-cobalt (http://www.ncbi.nlm.nih.gov/tools/cobalt/). PDZ domain prediction was performed using the ExPaSy Prosite tool ([75], http://prosite.expasy.org/). ExPaSy-Marcoil tool was used to predict coiled-coil domains ([76], http://bcf.isb-sib.ch/webmarcoil/webmarcoilC1.html). *C*. *elegans* gene expression was assessed using NEXTDB (nematode.lab.nig.ac.jp/) and [28,30,31].

### Chromosome movement assessment by live imaging

Wild-type and *gras-1* hermaphrodites carrying the *oxls279[Ppie-1*::*GFP*::*H2B*, *unc +](II); ieSi21 [sun-1p*::*sun-1*::*mRuby*::*sun-1 3’UTR + Cbr-unc-119(+)] IV* constructs were grown at 20°C or 25°C and selected at the L4 stage. 14-16h post-L4 live worms were mounted on 2% agarose pads with M9 containing 0.01% levamisole. Hyperstack images (x, y, z, t) at 594nm for SUN-1::mRuby fluorescence, were taken using the 60x or 100X objective at 0.2μm intervals. Nuclei with chromatin in the crescent-shaped configuration characteristic of the leptotene/zygotene stage were imaged every 5 seconds for a minute and SUN-1 aggregate trajectory was followed. An additional stack (x, y, z) capturing GFP::H2B signal at 523nm was collected as a reference for the chromatin shape. Images were registered using the Fiji (NIH) plugin Manual Registration. 2D speed analysis of SUN-1::mRuby aggregates was performed using the Fiji Manual Tracking plugin as in [17,18].

### Ethics statement

All mice used were bred at the Johns Hopkins University Bloomberg School of Public Health (JHSPH, Baltimore, MD) in accordance with criteria established by the National Institute of Health and the U.S. Department of Agriculture. The Johns Hopkins University Institutional Animal Care and Use Committee (IACUC) approved the protocols for the mice’s care and use.

### Mice mutant lines

*Tamalin* KO mice were kindly provided by Dr. Lino Tessarollo [58]. We obtained mice harboring the *Cytip* tm1a “knockout first” allele from the Mutant Mouse Resource and Research Centers (MMRRC) at University of California-Davis. The *Cytip* tm1a allele has loxP sites flanking exon 4 and 5. Mice heterozygous for *Cytip* tm1a were bred with mice harboring the *Spo11-Cre* transgene (C57BL/6-Tg Spo11-cre)Rsw/PecoJ), which express Cre recombinase in spermatocytes shortly after meiotic entry [77]https://sciwheel.com/work/citation?ids=1181041&pre=&suf=&sa=0&dbf=0. The resulting progeny from this cross harbored the *Cytip* tm1b KO allele. We subsequently bred mice harboring the Tamalin KO and Cytip KO alleles to create the *Tamalin*, *Cytip* DKO mice for analysis. We also bred mice heterozygous for *Cytip* tm1a allele with mice harboring FLP recombinase transgene (FLP tg/0) to produce progeny with the *Cytip* tm1c “conditional knockout” (cKO) allele. These mice were used to create the *Cytip* cKO mice that were homozygous for the *Cytip* tm1c allele and hemizygous for the *Spo11-Cre* transgene.

### Mouse genotyping

Mouse genotypes were obtained by polymerase chain reaction (PCR). Mice toe tips were digested in 50 mM NaOH at 95°C for 15 mins and 1M Tris-HCl pH 7.5 was added to the digestion. The digested toe tips were used as the DNA template in the PCR. Primers used in the PCRs are listed in S3 Table. PCR conditions: 90°C for 2 min, 30 cycles of 90°C for 20 s, 58°C for 30 s, 72°C for 1 min. PCR products were analyzed using 2% agarose gels.

### Histological analysis and tubule squash preparations

Testes were fixed in Bouins fixative, embedded in paraffin, and serial sections of 5-μm thickness were placed onto slides and stained with hematoxylin and eosin (H&E). Mouse tubule squashes were prepared as described in [78].

### Mouse chromatin spread preparations and imaging

Spermatocyte and oocyte chromatin spreads were prepared as previously described [78–80]. Primary antibodies and dilution used for immunolabeling are presented in S4 Table. Secondary antibodies against human, rabbit, rat, mouse, and guinea pig IgG and conjugated to Alexa 350, 488, 568, or 633 (Life Technologies) were used at a 1:500 dilution.

Images from chromatin spread preparations were captured using a Zeiss CellObserver Z1 microscope linked to an ORCA-Flash 4.0 CMOS camera (Hamamatsu). Testis sections stained with H&E staining were captured using a Zeiss AxioImager A2 microscope linked to an AxioCam ERc5s camera, or Keyence BZ-X800 fluorescence microscope. Images were analyzed and processed using ZEN 2012 blue edition imaging software (Zeiss) or with BZ-X800 Viewer and Analyzer software (Keyence).

### Statistical methods

The average of the data was used as a typical representation throughout the manuscript, accompanied by the standard error as a measure of data deviation. Statistical tests were performed in GraphPad Prism 8. Variables with continuous data, such as speed, distance, and area, were compared using unpaired 2-tailed t-tests. The Fisher exact test was used to assess the statistical significance for the distribution of data in the samples. Other comparisons were made using either the two-sided non-parametric Mann Whitney U-test or the Kruskall-Wallis test. Graphs for comparisons were generated in Microsoft Excel or GraphPad Prism 8.

## Supporting information

S1 Fig Expression of MmCytip, MmGrasp, and *Cegras-1* during spermatogenesis, and analysis of GRAS-1 localization. (**A**) Expression of Mus musculus Cytip and Grasp during the first wave of spermatogenesis via RT-PCR. (**B**) Expression of *C. elegans gras-1* throughout the germline (zones 1–10) as described in [31]. (**C**) GRAS-1::GFP localization in whole mounted gonads of *gras-1::gfp* male *C. elegans* by co-staining with anti-GFP (green) and DAPI (blue). (**D**) GRAS-1::GFP expression in live whole worms. Shown are differential interference contrast (DIC; top) and GFP (bottom) images, of wild type and *gras-1::gfp* worms. Heads are indicated by asterisks. Gonads in areas marked by yellow dashed rectangles are magnified. GFP signal detected in the intestine is due to autofluorescence and is also observed in wild type. GRAS-1::GFP expression is only clearly detected in the gonad. (**E**) Representative image of the early pachytene region in *gras-1::gfp* hermaphrodites co-stained for GRAS-1::GFP (green), Phalloidin (red), SYX-4 (white) and DAPI (blue). (**F**) Representative image of early pachytene region in *gras-1::gfp* hermaphrodites co-stained for GRAS-1::GFP (green), Tubulin (magenta) and DAPI (blue).
(TIF)

S2 Fig Intensity correlation analysis for GRAS-1::GFP and S8 pSUN-1, analysis of GRAS-1::GFP localization dependence on DSB formation and synapsis, and *gras-1* mutant phenotypes. (**A**) Left, representative line-plot of S8 pSUN-1 (red) and GRAS-1::GFP (green) signal intensities along the region of the nuclear envelope adjacent to clustered chromosomes in leptotene/zygotene. The data represent a positive correlation of 0.454 by the Pearson correlation coefficient. Right, representative image of selection of a region of interest (outlined in white and indicated by white arrow) for intensity correlation analysis of a leptotene/zygotene nucleus. (**B**) GRAS-1::GFP localization in whole mounted gonads of *gras-1::gfp* (wt, top), *gras-1::gfp;spo-11* (middle) and *gras-1::gfp;syp-2* (bottom) hermaphrodite *C. elegans* by co-immunostaining with anti-GFP (green) and DAPI (blue). GRAS-1::GFP signal alone is shown in white. Regions of the gonads indicated by yellow dashed rectangles and shown in higher magnification to the right (oriented top to bottom) encompass nuclei in the premeiotic tip, leptotene/zygotene, and early pachytene stages. (**C**) The mean number of eggs laid (brood size) ± SEM, as well as the percentage of embryonic lethality, larval lethality, and males are shown for the indicated genotypes. *p < 0.05, **p < 0.01, ****p < 0.0001 by Fisher’s exact test. n = number of worms for which entire broods were analyzed in at least two independent biological replicates.
(TIF)

S3 Fig Switch in PLK-2 localization and SC central region formation are GRAS-1-dependent, but SC defects are IMA-1 and IMA-2-independent. (**A**) High-resolution images of leptotene/zygotene and mid pachytene nuclei from wild type and *gras-1* germlines stained with anti-PLK-2 (magenta) and DAPI (blue). n = 10 and 7 gonads each. (**B**) Left, high-resolution images of pachytene nuclei in wild type and *gras-1* germlines co-stained with anti-SYP-1 (magenta), anti-HTP-3 (green) and DAPI (blue). Yellow arrows indicate nuclei with SYP-1 discontinuities. Right, histogram showing the frequency of nuclei found with 0–4 SYP-1 discontinuities during mid pachytene stage in wild type and *gras-1*. n = 118 and 178, respectively, p = 0.002, Fisher’s Exact test. (**C**) High-resolution images of mid pachytene stage nuclei from the indicated genotypes co-stained with anti-SYP-1 (magenta), anti-REC-8 (green) and DAPI (blue). All images are from at least two independent biological replicates.
(TIF)

S4 Fig GRAS-1 interacts with SC central region proteins SYP-1, SYP-2, and SYP-3, and contributes to SPO-11-dependent DSB repair. **(A)** Western blot using an anti-SYP-3 antibody showing immunoprecipitation of SYP-3 from *gras-1*::*gfp* and *syp-2*::*gfp* (positive control) but not wild type (negative control) whole worm lysates done with a GFP antibody. **(B)** The yeast two-hybrid system was used to examine the protein interactions between GRAS-1 full length, ΔNt^69-245^ and ΔCt^1-163^ truncations, and SYP-1/2/3/4 full length, N-terminal, and C-terminal truncations (AD, activation domain; DB, Gal4 DNA binding domain). Negative (no. 1) and positive controls (nos. 2–4) were used as described in [63]. SYP-3 ΔC, SYP-4 full length and SYP-4 ΔN exhibited strong self-activation and therefore their observed interactions are false positives. **(C)** Live imaging of DHC-1::GFP (green) and TIR1::mRuby (red) proteins in leptotene/zygotene nuclei *of dhc-1*::*AID*::*gfp;ieSi38* worms grown in bacteria expressing either the empty vector or *dhc-1(RNAi)*. **(D)** Left, high-resolution images of mid-pachytene nuclei in *spo-11* and *gras-1;spo-11* stained with anti-RAD-51 (green) and DAPI (blue). Right, Histogram showing the mean number of RAD-51 foci/nucleus scored along the germlines of the indicated genotypes. 6 gonads were scored per genotype in two independent biological replicates. Error bars represent the SEM. Not significant by the Mann-Whitney U-test. **(E)** Histogram showing the mean number of germ cell corpses in wild type, *spo-11*, *gras-1*, and *gras-1;spo-11* worms. Error bars represent the SEM. ****p<0.0001, Mann-Whitney U-test, n = 39, 40, 30 and 48 gonads, respectively.
(TIF)

S5 Fig Tamalin-Cytip DKO male mouse analysis. (**A**) Representative structure of *Cytip* tm1a and tm1b *Mus musculus* alleles. Numbered exons shown in grey boxes, FRT: flippase recognition target, lacZ reporter, loxP: locus of X-over P1 site, neo: neomycin resistance gene. (**B**) Cross section of testis (top panel) and epididymitis (bottom panel) of control (301dpp) and Tamalin-Cytip DKO (196dpp) mice stained with hematoxylin and eosin. Graph shows no significant difference in testis weight from control and Tamalin-Cytip DKO. Error bars show mean ± SEM. Two-tailed Student’s t-test, n = 3 mice each. (**C**) Chromatin spreads from early meiotic prophase (zygonema), mid meiotic prophase (pachynema) and late meiotic prophase (diplonema) control and Tamalin-Cytip DKO *Mus musculus* spermatocytes co-immunostained with antibodies against SYCP3 (magenta) and γ-H2AX (green). Insets show normal chromatin morphology (DAPI). n = 50 cells per mouse and 3 mice per genotype. (**D**) Chromatin spreads from early and late zygonema, pachynema and diplonema cells of control and Tamalin-Cytip DKO *Mus musculus* spermatocytes co-immunostained with antibodies against SYCP3 (magenta) and ATR (green). Insets show normal chromatin morphology (DAPI). (**E**) Chromatin spreads from early and late zygonema, pachynema and diplonema cells of control and Tamalin-Cytip DKO Mus musculus spermatocytes co-immunostained with antibodies against SYCP3 (magenta) and RPA (green). Insets show normal chromatin morphology (DAPI).
(TIF)

S6 Fig Tamalin-Cytip DKO female mouse analysis and HsCYTIP complementation of *gras-1* mutants. (**A**) Chromatin spreads from pachynema and diplonema cells of control and Tamalin-Cytip DKO *Mus musculus* oocytes co-immunostained with antibodies against SYCP1 (green) and SYCP3 (magenta). Insets show normal chromatin morphology (DAPI). (**B**) Top, chromatin spreads from pachynema cells of control and Tamalin-Cytip DKO *Mus musculus* spermatocytes co-immunostained with antibodies against SYCP3 (magenta) and MLH1 (green). Insets show normal chromatin morphology (DAPI). Bottom, dot plot of the number of MLH1 foci per chromosome quantified in control and DKO oocytes. 21 cells per genotype, ns: not significant by Mann-Whitney U-test. (**C**) Top, chromatin spreads from pachynema cells of control and Tamalin-Cytip DKO *Mus musculus* oocytes co-immunostained with antibodies against SYCP3 (magenta) and CDK2 (green). Insets show normal chromatin morphology (DAPI). Bottom, dot plot of the number of CDK2 foci per chromosome quantified in control and DKO oocytes. 25 cells per genotype, ns: not significant by Mann-Whitney U-test. (**D**) Schematic representation of the genomic location of SKI LODGE germline insertion and the HsCYTIP complementation cassette. (**E**) RT-PCR analysis using primers specific for *gras-1, HsCYTIP*, and *gpdh-1* as a control shows *HsCYTIP* expression in the *gras-1;HsCYTIP* line and *gras-1* expression in wild type and *gras-1::gfp* lines. (**F**) Western blot showing HsCYTIP expression in lysates from *gras-1;HsCYTIP* worms and not in lysates from either wild type or *gras-1;empty* cassette worms, detected with anti-FLAG antibody. Anti-tubulin is used as loading control. (**G**) High-resolution images of whole mounted gonads of wild type, *gras-1;empty* and *gras-1;HsCYTIP* during early pachytene co-stained with anti-SYP-1 (magenta) and DAPI (blue). Yellow arrows indicate SYP-1 aggregates. Right, dot plot of the number of SYP-1 aggregates per gonad. 10 to 11 gonads scored per genotype. ***p<0.001 by Kruskall-Wallis test.
(TIF)

S1 Table List of *C. elegans* lines used in this study. (DOCX)

S2 Table Primary antibodies used for *C. elegans* immunostainings. (DOCX)

S3 Table Mouse genotyping primers and products. (DOCX)

S4 Table Primary and secondary antibodies used for mouse chromatin spreads. (DOCX)

S1 Data Raw data for analyses in main and supplemental figures. (XLSX)

S1 Video *gras-1* movement defects depend on dynein. (MP4)

S2 Video GRAS-1 phosphorylation affects its role in chromosome movement. (MP4)
