# Supplementary figures and images for: GRAS-1 is a novel regulator of early meiotic chromosome dynamics in C. elegans
Source: PLoS Genet. 2023 Feb 21;19(2):e1010666. doi: 10.1371/journal.pgen.1010666 (PMC9983901; doi:10.1371/journal.pgen.1010666)

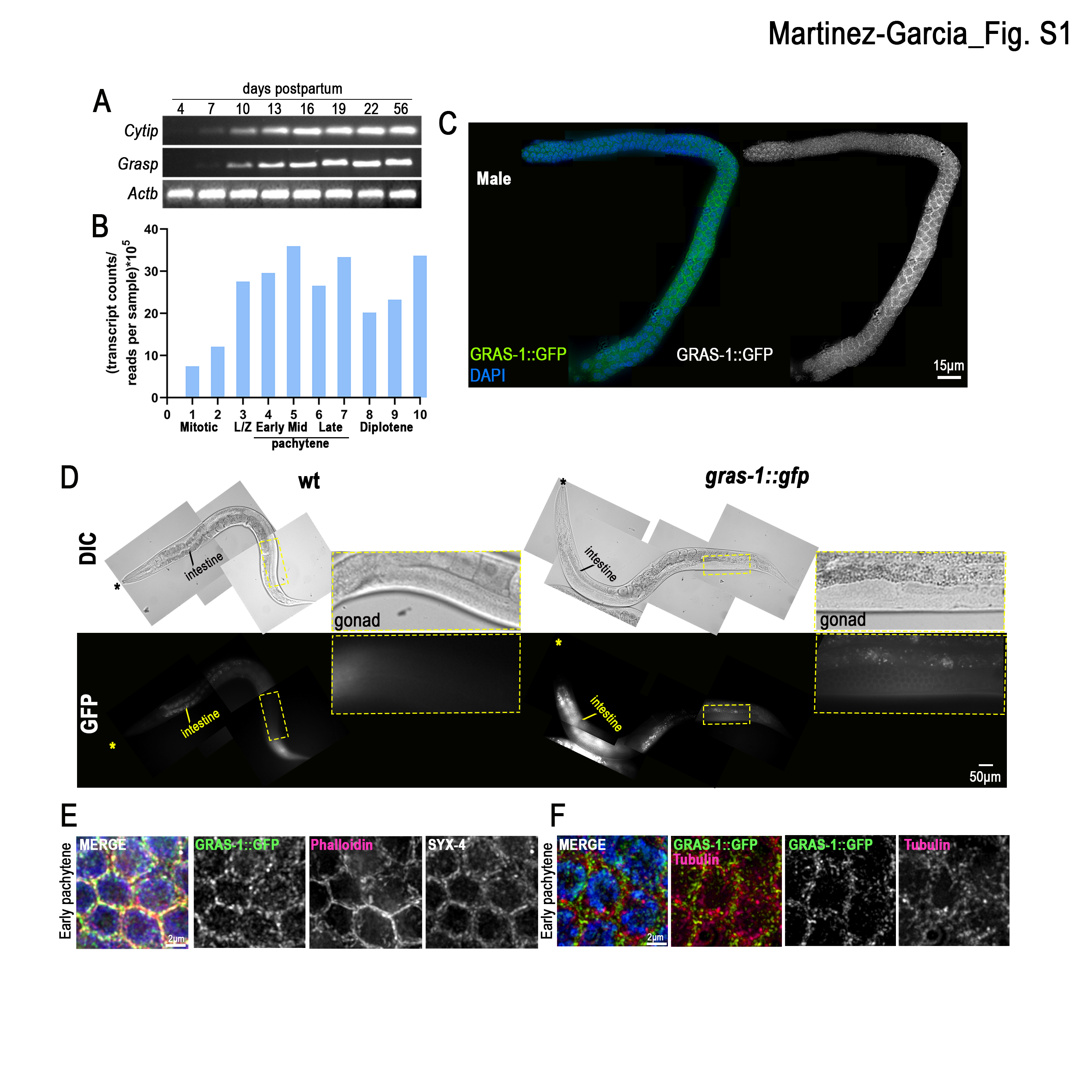

Supplement: S1 Fig — (A) Expression of Mus musculus Cytip and Grasp during the first wave of spermatogenesis via RT-PCR. (B) Expression of C. elegans gras-1 throughout the germline (zones 1–10) as described in [31]. (C) GRAS-1::GFP localization in whole mounted gonads of gras-1::gfp male C. elegans by co-staining with anti-GFP (green) and DAPI (blue). (D) GRAS-1::GFP expression in live whole worms. Shown are differential interference contrast (DIC; top) and GFP (bottom) images, of wild type and gras-1::gfp worms. Heads are indicated by asterisks. Gonads in areas marked by yellow dashed rectangles are magnified. GFP signal detected in the intestine is due to autofluorescence and is also observed in wild type. GRAS-1::GFP expression is only clearly detected in the gonad. (E) Representative image of the early pachytene region in gras-1::gfp hermaphrodites co-stained for GRAS-1::GFP (green), Phalloidin (red), SYX-4 (white) and DAPI (blue). (F) Representative image of early pachytene region in gras-1::gfp hermaphrodites co-stained for GRAS-1::GFP (green), Tubulin (magenta) and DAPI (blue). (TIF) [file pgen.1010666.s001.tif]

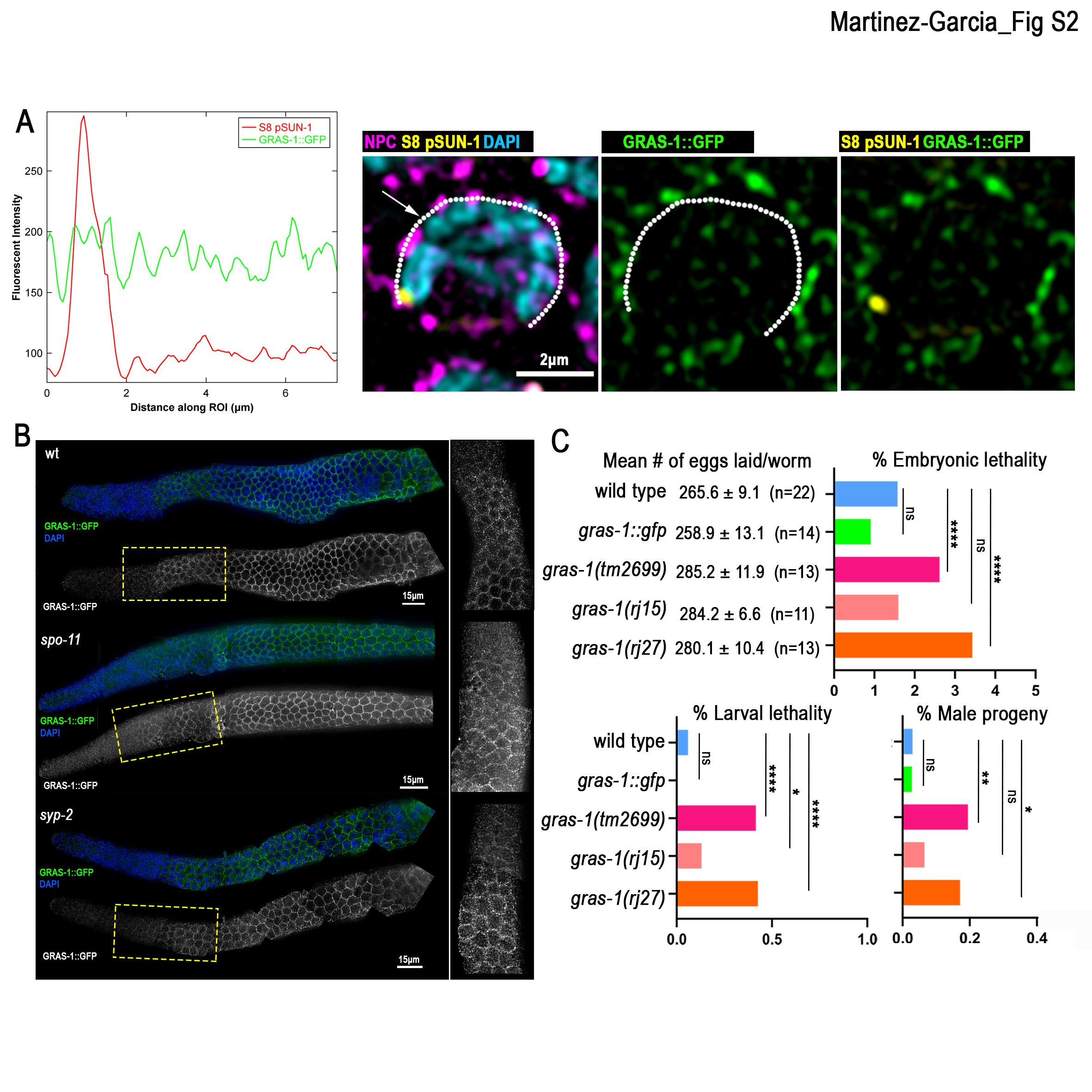

Supplement: S2 Fig — (A) Left, representative line-plot of S8 pSUN-1 (red) and GRAS-1::GFP (green) signal intensities along the region of the nuclear envelope adjacent to clustered chromosomes in leptotene/zygotene. The data represent a positive correlation of 0.454 by the Pearson correlation coefficient. Right, representative image of selection of a region of interest (outlined in white and indicated by white arrow) for intensity correlation analysis of a leptotene/zygotene nucleus. (B) GRAS-1::GFP localization in whole mounted gonads of gras-1::gfp (wt, top), gras-1::gfp;spo-11 (middle) and gras-1::gfp;syp-2 (bottom) hermaphrodite C. elegans by co-immunostaining with anti-GFP (green) and DAPI (blue). GRAS-1::GFP signal alone is shown in white. Regions of the gonads indicated by yellow dashed rectangles and shown in higher magnification to the right (oriented top to bottom) encompass nuclei in the premeiotic tip, leptotene/zygotene, and early pachytene stages. (C) The mean number of eggs laid (brood size) ± SEM, as well as the percentage of embryonic lethality, larval lethality, and males are shown for the indicated genotypes. *p < 0.05, **p < 0.01, ****p < 0.0001 by Fisher’s exact test. n = number of worms for which entire broods were analyzed in at least two independent biological replicates. (TIF) [file pgen.1010666.s002.tif]

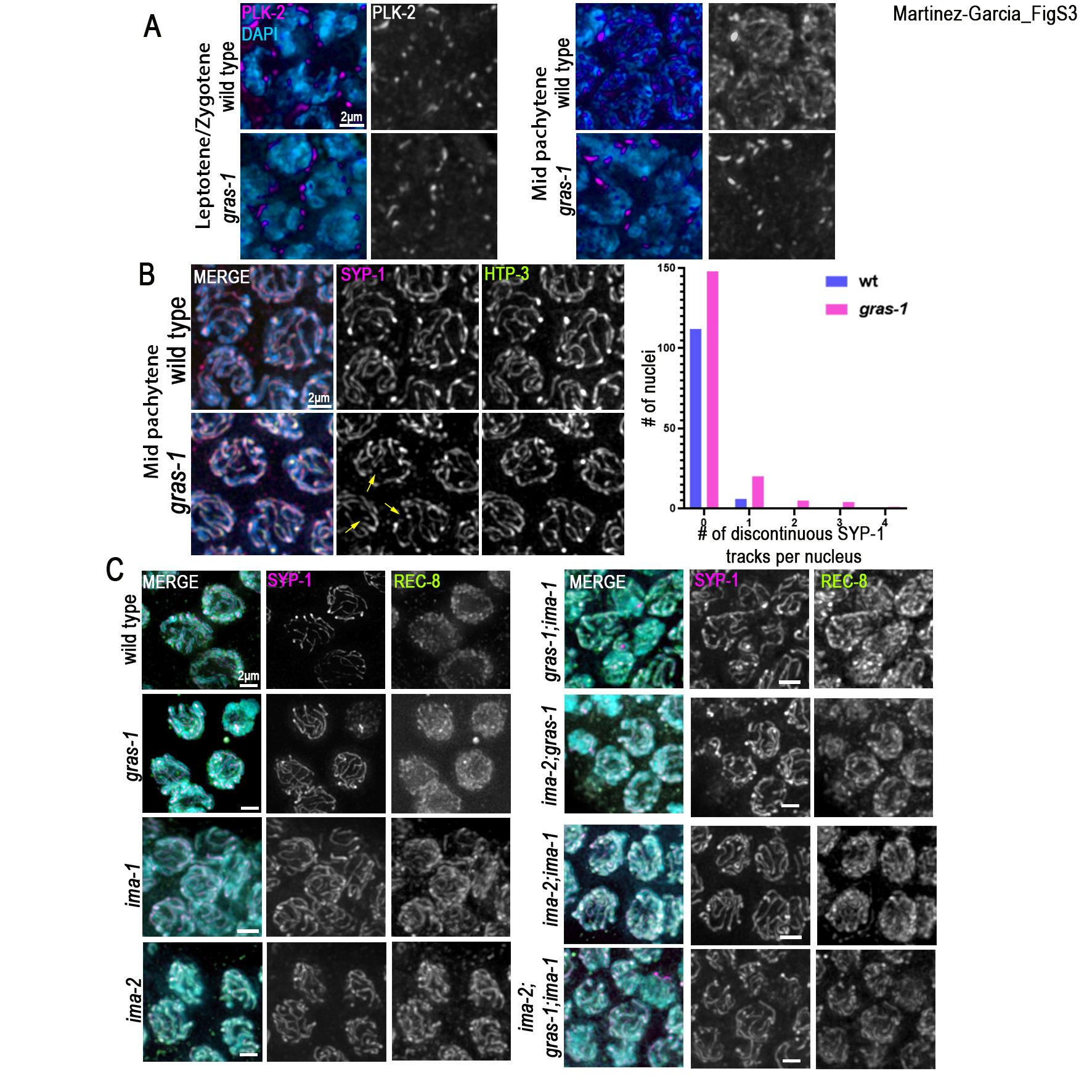

Supplement: S3 Fig — (A) High-resolution images of leptotene/zygotene and mid pachytene nuclei from wild type and gras-1 germlines stained with anti-PLK-2 (magenta) and DAPI (blue). n = 10 and 7 gonads each. (B) Left, high-resolution images of pachytene nuclei in wild type and gras-1 germlines co-stained with anti-SYP-1 (magenta), anti-HTP-3 (green) and DAPI (blue). Yellow arrows indicate nuclei with SYP-1 discontinuities. Right, histogram showing the frequency of nuclei found with 0–4 SYP-1 discontinuities during mid pachytene stage in wild type and gras-1. n = 118 and 178, respectively, p = 0.002, Fisher’s Exact test. (C) High-resolution images of mid pachytene stage nuclei from the indicated genotypes co-stained with anti-SYP-1 (magenta), anti-REC-8 (green) and DAPI (blue). All images are from at least two independent biological replicates. (TIF) [file pgen.1010666.s003.tif]

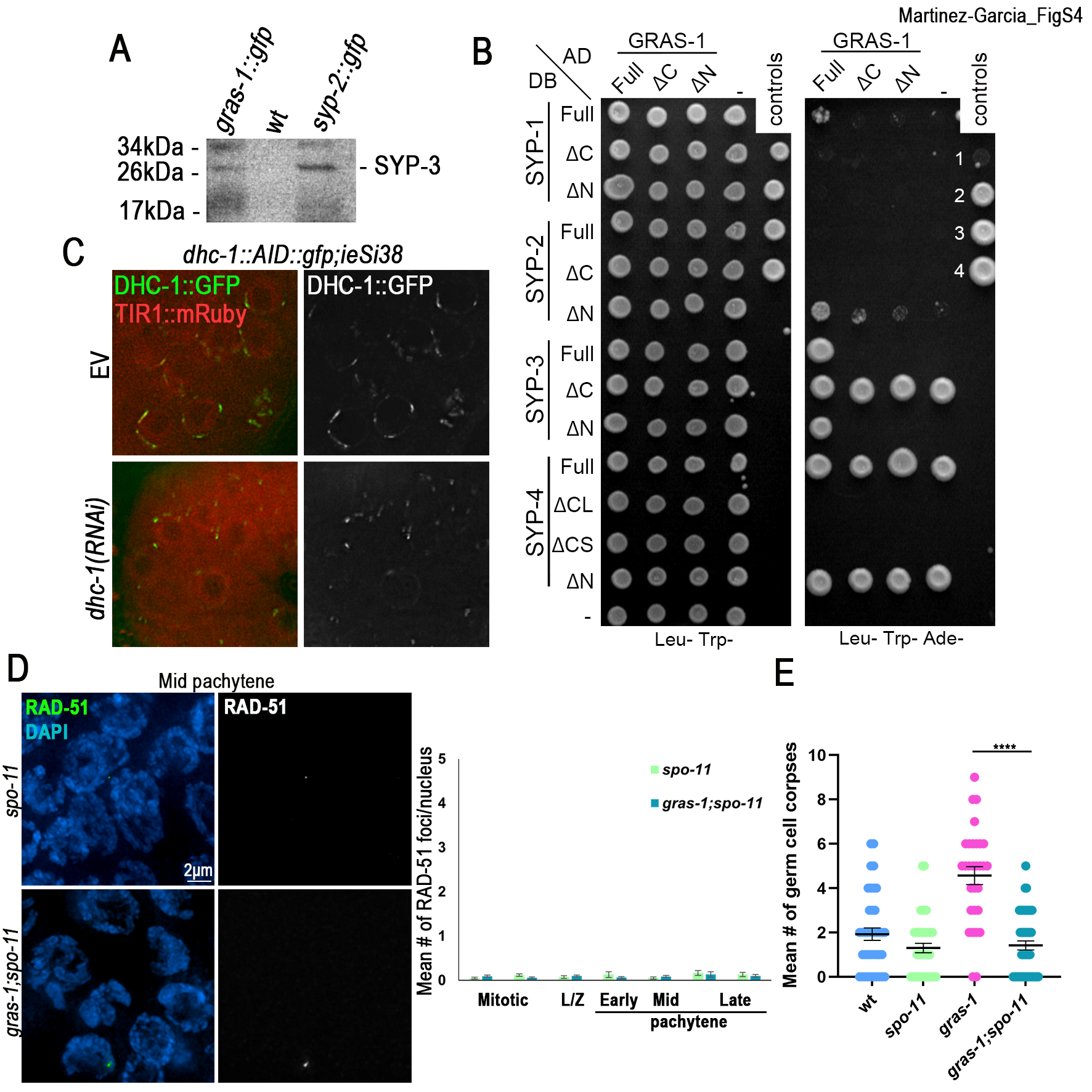

Supplement: S4 Fig — (A) Western blot using an anti-SYP-3 antibody showing immunoprecipitation of SYP-3 from gras-1::gfp and syp-2::gfp (positive control) but not wild type (negative control) whole worm lysates done with a GFP antibody. (B) The yeast two-hybrid system was used to examine the protein interactions between GRAS-1 full length, ΔNt69-245 and ΔCt1-163 truncations, and SYP-1/2/3/4 full length, N-terminal, and C-terminal truncations (AD, activation domain; DB, Gal4 DNA binding domain). Negative (no. 1) and positive controls (nos. 2–4) were used as described in [63]. SYP-3 ΔC, SYP-4 full length and SYP-4 ΔN exhibited strong self-activation and therefore their observed interactions are false positives. (C) Live imaging of DHC-1::GFP (green) and TIR1::mRuby (red) proteins in leptotene/zygotene nuclei of dhc-1::AID::gfp;ieSi38 worms grown in bacteria expressing either the empty vector or dhc-1(RNAi). (D) Left, high-resolution images of mid-pachytene nuclei in spo-11 and gras-1;spo-11 stained with anti-RAD-51 (green) and DAPI (blue). Right, Histogram showing the mean number of RAD-51 foci/nucleus scored along the germlines of the indicated genotypes. 6 gonads were scored per genotype in two independent biological replicates. Error bars represent the SEM. Not significant by the Mann-Whitney U-test. (E) Histogram showing the mean number of germ cell corpses in wild type, spo-11, gras-1, and gras-1;spo-11 worms. Error bars represent the SEM. ****p<0.0001, Mann-Whitney U-test, n = 39, 40, 30 and 48 gonads, respectively. (TIF) [file pgen.1010666.s004.tif]

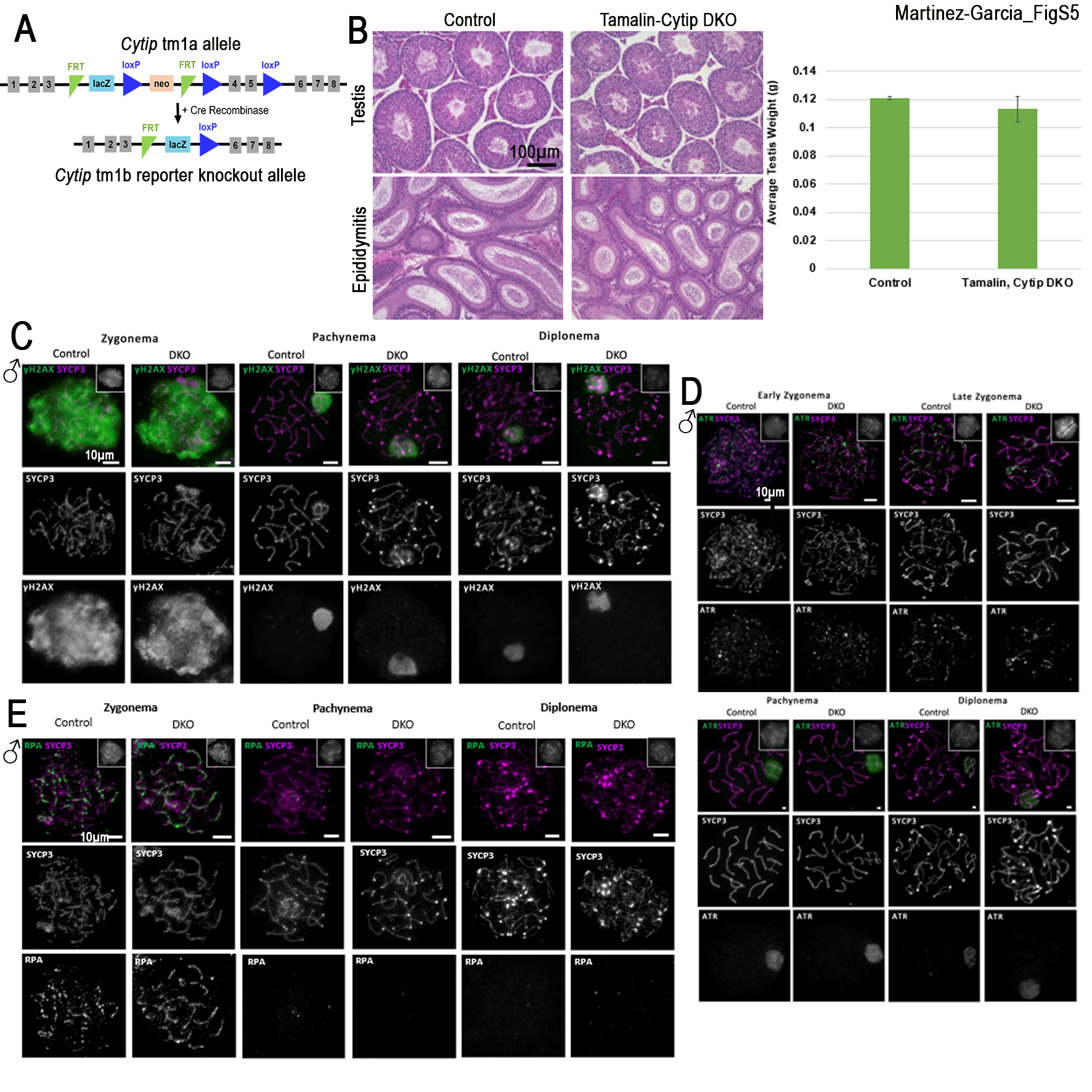

Supplement: S5 Fig — (A) Representative structure of Cytip tm1a and tm1b Mus musculus alleles. Numbered exons shown in grey boxes, FRT: flippase recognition target, lacZ reporter, loxP: locus of X-over P1 site, neo: neomycin resistance gene. (B) Cross section of testis (top panel) and epididymitis (bottom panel) of control (301dpp) and Tamalin-Cytip DKO (196dpp) mice stained with hematoxylin and eosin. Graph shows no significant difference in testis weight from control and Tamalin-Cytip DKO. Error bars show mean ± SEM. Two-tailed Student’s t-test, n = 3 mice each. (C) Chromatin spreads from early meiotic prophase (zygonema), mid meiotic prophase (pachynema) and late meiotic prophase (diplonema) control and Tamalin-Cytip DKO Mus musculus spermatocytes co-immunostained with antibodies against SYCP3 (magenta) and γ-H2AX (green). Insets show normal chromatin morphology (DAPI). n = 50 cells per mouse and 3 mice per genotype. (D) Chromatin spreads from early and late zygonema, pachynema and diplonema cells of control and Tamalin-Cytip DKO Mus musculus spermatocytes co-immunostained with antibodies against SYCP3 (magenta) and ATR (green). Insets show normal chromatin morphology (DAPI). (E) Chromatin spreads from early and late zygonema, pachynema and diplonema cells of control and Tamalin-Cytip DKO Mus musculus spermatocytes co-immunostained with antibodies against SYCP3 (magenta) and RPA (green). Insets show normal chromatin morphology (DAPI). (TIF) [file pgen.1010666.s005.tif]

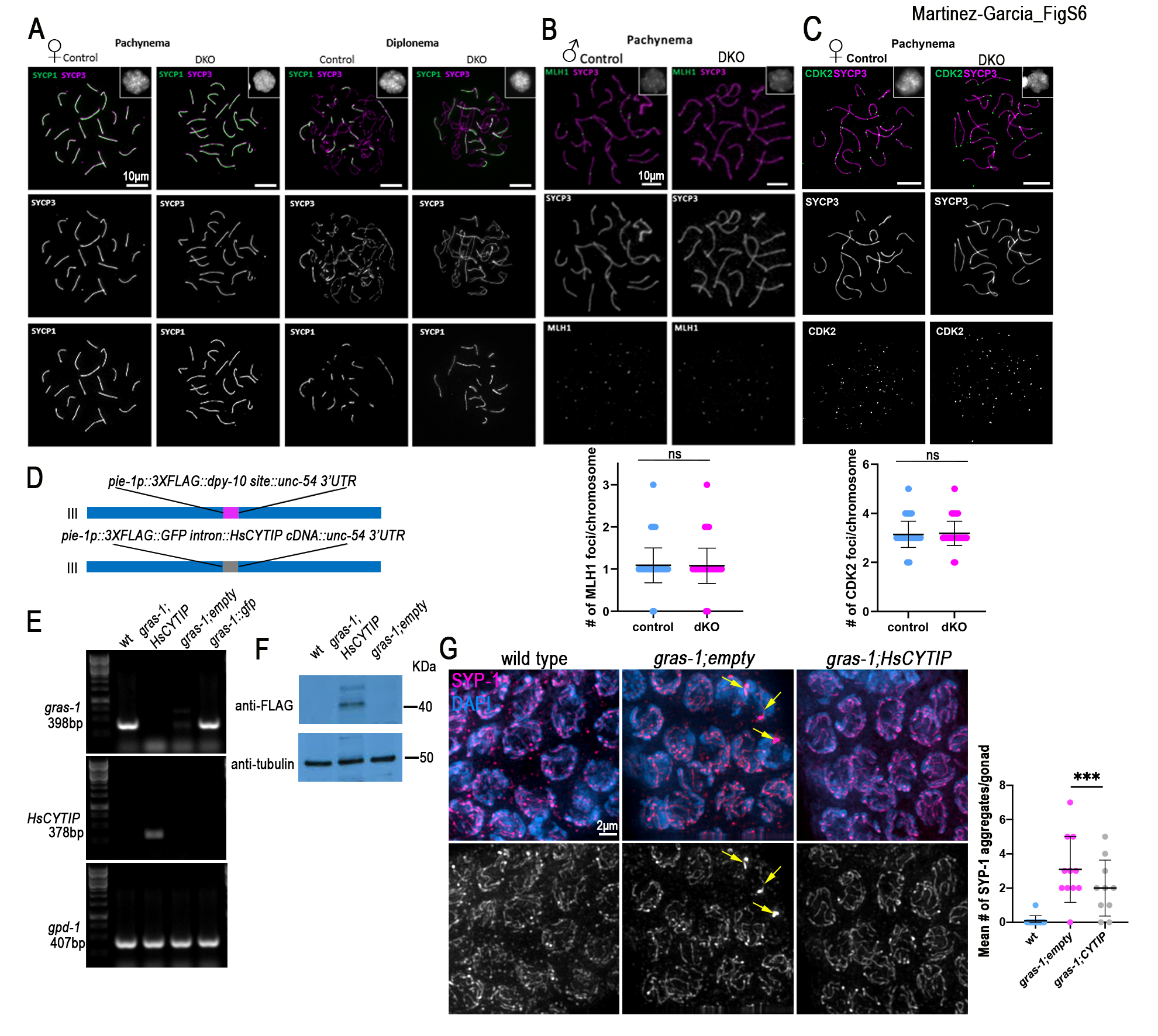

Supplement: S6 Fig — (A) Chromatin spreads from pachynema and diplonema cells of control and Tamalin-Cytip DKO Mus musculus oocytes co-immunostained with antibodies against SYCP1 (green) and SYCP3 (magenta). Insets show normal chromatin morphology (DAPI). (B) Top, chromatin spreads from pachynema cells of control and Tamalin-Cytip DKO Mus musculus spermatocytes co-immunostained with antibodies against SYCP3 (magenta) and MLH1 (green). Insets show normal chromatin morphology (DAPI). Bottom, dot plot of the number of MLH1 foci per chromosome quantified in control and DKO oocytes. 21 cells per genotype, ns: not significant by Mann-Whitney U-test. (C) Top, chromatin spreads from pachynema cells of control and Tamalin-Cytip DKO Mus musculus oocytes co-immunostained with antibodies against SYCP3 (magenta) and CDK2 (green). Insets show normal chromatin morphology (DAPI). Bottom, dot plot of the number of CDK2 foci per chromosome quantified in control and DKO oocytes. 25 cells per genotype, ns: not significant by Mann-Whitney U-test. (D) Schematic representation of the genomic location of SKI LODGE germline insertion and the HsCYTIP complementation cassette. (E) RT-PCR analysis using primers specific for gras-1, HsCYTIP, and gpdh-1 as a control shows HsCYTIP expression in the gras-1;HsCYTIP line and gras-1 expression in wild type and gras-1::gfp lines. (F) Western blot showing HsCYTIP expression in lysates from gras-1;HsCYTIP worms and not in lysates from either wild type or gras-1;empty cassette worms, detected with anti-FLAG antibody. Anti-tubulin is used as loading control. (G) High-resolution images of whole mounted gonads of wild type, gras-1;empty and gras-1;HsCYTIP during early pachytene co-stained with anti-SYP-1 (magenta) and DAPI (blue). Yellow arrows indicate SYP-1 aggregates. Right, dot plot of the number of SYP-1 aggregates per gonad. 10 to 11 gonads scored per genotype. ***p<0.001 by Kruskall-Wallis test. (TIF) [file pgen.1010666.s006.tif]
