# Supplementary material for: GRAS-1 is a novel regulator of early meiotic chromosome dynamics in C. elegans
Source: PLoS Genet. 2023 Feb 21;19(2):e1010666. doi: 10.1371/journal.pgen.1010666 (PMC9983901; doi:10.1371/journal.pgen.1010666)
Supplement: S1 Table — (DOCX) [file pgen.1010666.s007.docx]

**Supplemental table 1. List of *C. elegans* lines used in this study**

| **Strain name** | **Genotype** | **Reference** |
| --- | --- | --- |
| **PHX598** | *gras-1::gfp(syb598[gras-1::gfp])(I)* | This study |
| **CV392** | *gras-1(rj15) I/hT2[bli-4(e937) let-? (q782) qls48] (I:III)* | This study |
| **CV464** | *gras-1(rj27) I/hT2[bli-4(e937) let-? (q782) qls48] (I:III)* | This study |
| **CV464** | *gras-1(rj28) I/hT2[bli-4(e937) let-? (q782) qls48] (I:III)* | This study |
| **CV826** | *gras-1(syb1380)/hT2[bli-4(e937) let-?(q782) qls48] (I;III)* | This study |
| **CV849** | *gras-1(syb1899)/hT2[bli-4(e937) let-?(q782) qls48] (I;III)* | This study |
| **AV106** | *spo-11 (ok79) IV/nT1 [unc-? (n754) let-?] (IV;V)* | (1) |
| **CV819** | *gras-1(rj28) I/hT2[bli-4(e937) let-? (q782) qls48] (I:III); spo-11 (ok79) IV/nT1 [unc-? (n754) let-?] (IV;V)* | This study |
| **CV835** | *oxls279[Ppie-1::GFP::H2B, unc +](II); ieSi21 [sun-1p::sun-1::mRuby::sun-1 3'UTR + Cbr-unc-119(+)] IV.* | This study; (2,3) |
| **CV832** | *gras-1(rj28) I/hT2[bli-4(e937) let-? (q782) qls48] (I:III); oxls279[Ppie-1::GFP::H2B, unc +](II); ieSi21 [sun-1p::sun-1::mRuby::sun-1 3'UTR + Cbr-unc-119(+)] IV.* | This study |
| **CV812** | *gras-1::gfp(syb598[gras-1::gfp])(I);* *syp-2 (ok307) V/ nT1[Unc-? (n754) let-? qIs 50] (IV;V)* | This study; (4) |
| **CV818** | *gras-1::gfp(syb598[gras-1::gfp])(I); spo-11 (ok79) IV/nT1 [unc-? (n754) let-?] (IV;V)* | This study |
| **CV821** | *gras-1::gfp(syb598[gras-1::gfp])(I); chk-2(ok3037)* | This study; VC3236 |
| **WBM1119** | *wbmls60[pie-1p::3xFLAG::dpy-10 crRNA::unc-54 3’UTR, III]* | (5) |
| **CV882** | *gras-1(rj28) (I); wbmls60[pie-1p::3xFLAG::dpy-10 crRNA::unc-54 3’UTR, III]* | This study |
| **CV870** | *gras-1(rj28) (I); rj55[pie-1p::3XFLAG::HsCYTIP::unc-54 3’UTR, III]* | This study |
| **CA1199** | *ieSi38 [sun-1p::TIR1::mRuby::sun-1 3'UTR + Cbr-unc-119(+)] IV* | (6) |
| **CA1215** | *dhc-1(ie28[dhc-1::degron::GFP]) I; ieSi38 [sun-1p::TIR1::mRuby::sun-1 3'UTR + Cbr-unc-119(+)] IV* | (6) |

References

1. Dernburg AF, McDonald K, Moulder G, Barstead R, Dresser M, Villeneuve AM. Meiotic recombination in *C. elegans* initiates by a conserved mechanism and is dispensable for homologous chromosome synapsis. Cell. 1998 Aug;94(3):387–98.

2. Frøkjær-Jensen C, Wayne Davis M, Hopkins CE, Newman BJ, Thummel JM, Olesen SP, et al. Single-copy insertion of transgenes in *Caenorhabditis elegans*. Nat Genet. 2008 Nov;40(11):1375–83.

3. Rog O, Dernburg AF. Direct visualization reveals kinetics of meiotic chromosome synapsis. Cell Reports. 2015 Mar;10(10):1639–45.

4. Colaiácovo MP, MacQueen AJ, Martinez-Perez E, McDonald K, Adamo A, La Volpe A, et al. Synaptonemal complex assembly in *C. elegans* is dispensable for loading strand-exchange proteins but critical for proper completion of recombination. Dev Cell. 2003 Sep;5(3):463–74.

5. Silva-García CG, Lanjuin A, Heintz C, Dutta S, Clark NM, Mair WB. Single-Copy Knock-In Loci for Defined Gene Expression in *Caenorhabditis elegans*. G3. 2019 Jul;9(7):2195–8.

6. Zhang L, Ward JD, Cheng Z, Dernburg AF. The auxin-inducible degradation (AID) system enables versatile conditional protein depletion in *C. elegans*. Development. 2015 Dec 15;142(24):4374–84.
