# Supplementary material for: GRAS-1 is a novel regulator of early meiotic chromosome dynamics in C. elegans
Source: PLoS Genet. 2023 Feb 21;19(2):e1010666. doi: 10.1371/journal.pgen.1010666 (PMC9983901; doi:10.1371/journal.pgen.1010666)
Supplement: S2 Table — (DOCX) [file pgen.1010666.s008.docx]

**Supplemental table 2. Primary antibodies used for *C. elegans* immunostainings.**

| **Antibody** | **Host** | **Dilution** | **Reference** |
| --- | --- | --- | --- |
| α-GFP | Chicken | 1:500 | ab13970, Abcam |
| α-RFP | Rabbit | 1:100 | ab62341, Abcam |
| α-pSer8 SUN-1 | Guinea Pig | 1:700 | (1) |
| α-HIM-8 | Rabbit | 1:500 | 41980002, Novus Biological - SDI |
| α-RAD-51 | Rabbit | 1:10,000 | 29480002, Novus Biological - SDI |
| α-HTP-3 | Guinea Pig | 1:500 | (2) |
| α-SYP-1 | Goat | 1:2,000 | (3) |
| α-PLK-2 | Rabbit | 1:200 | (4) |
| α-REC-8 | Rabbit | 1:500 | SDQ0802, Novus Biologicals |
| α-SYX-4 | Rabbit | 1:300 | (5) |
| Phalloidin-Atto 488 | - | 1:400 | 49409, Sigma Aldrich |
| α-HIM-3 | Chicken | 1:400 | Gift from M. Zetka (2) |
| α-ZHP-3 | Guinea Pig | 1:500 | (6) |
| α-NPC | Mouse | 1:500 | Mab414 (ab24609), Abcam |
| α-tubulin | Mouse | 1:200 | T9026, Sigma-Aldrich |

References

1. Woglar A, Daryabeigi A, Adamo A, Habacher C, Machacek T, La Volpe A, et al. Matefin/SUN-1 phosphorylation is part of a surveillance mechanism to coordinate chromosome synapsis and recombination with meiotic progression and chromosome movement. PLoS Genet. 2013 Mar 7;9(3):e1003335.

2. Goodyer W, Kaitna S, Couteau F, Ward JD, Boulton SJ, Zetka M. HTP-3 links DSB formation with homolog pairing and crossing over during *C. elegans* meiosis. Developmental Cell. 2008 Feb;14(2):263–74.

3. Nadarajan S, Lambert TJ, Altendorfer E, Gao J, Blower MD, Waters JC, et al. Polo-like kinase-dependent phosphorylation of the synaptonemal complex protein SYP-4 regulates double-strand break formation through a negative feedback loop. eLife. 2017 Mar 27;6:e23437.

4. Nishi Y, Rogers E, Robertson SM, Lin R. Polo kinases regulate *C. elegans* embryonic polarity via binding to DYRK2-primed MEX-5 and MEX-6. Development. 2008 Feb 15;135(4):687–97.

5. Jantsch-Plunger V, Glotzer M. Depletion of syntaxins in the early *Caenorhabditis elegans* embryo reveals a role for membrane fusion events in cytokinesis. Curr Biol. 1999 Jul 15;9(14):738–45.

6. Bhalla N, Wynne DJ, Jantsch V, Dernburg AF. ZHP-3 acts at crossovers to couple meiotic recombination with synaptonemal complex disassembly and bivalent formation in *C. elegans*. PLoS Genet. 2008 Oct;4(10):e1000235.
