# Supplementary material for: GRAS-1 is a novel regulator of early meiotic chromosome dynamics in C. elegans
Source: PLoS Genet. 2023 Feb 21;19(2):e1010666. doi: 10.1371/journal.pgen.1010666 (PMC9983901; doi:10.1371/journal.pgen.1010666)
Supplement: S3 Table — (DOCX) [file pgen.1010666.s009.docx]

**Supplemental table 3. Mouse genotyping primers and products**

| ***Gene*** | **Forward Primer**  **(5'-…-3')** | **Reverse Primer**  **(5'-...-3')** | **Band Size (bp)** |
| --- | --- | --- | --- |
| *Spo11-Cre* Transgene | CCATCTGCCA  CCAGCCAG | TCGCCATCTTC  CAGCAGG | 281 |
| *Cre* Internal Control (Cpxm1) | ACTGGGATCTTCG  AACTCTTTGGAC | GATGTTGGGGCA  CTGCTCATTCACC | 420 |
| *Tamalin* WT | CTGCTTGCAGGTTT  CCACAGCTTC | CTACAGCCTTCTGA  GACCCGAGTG | 330 |
| *Tamalin KO* | CTGCTTGCAGGTT  TCCACAGCTTC | GAATGATGGCCTT  AGTGGTTCGTG | 436 |
| *Cytip tm1b* | GCTACCATTACCAGTTGGTCTGGTGTC | TGAGTAGCTGGGA  AGACCAATGTCC | 676 |
| *Cytip WT, Cytip tm1c* | CAATCCCACCAGA  TCATCAACAGCC | ACTGATCATCCTGTCTCAGGTGTGG | 719, 861 |
| *Cytip tm1c* | GAGATGGCGCAACGCAATTAATG | TGAGTAGCTGGGAAGACCAATGTCC | 335 |
