# Supplementary material for: GRAS-1 is a novel regulator of early meiotic chromosome dynamics in C. elegans
Source: PLoS Genet. 2023 Feb 21;19(2):e1010666. doi: 10.1371/journal.pgen.1010666 (PMC9983901; doi:10.1371/journal.pgen.1010666)
Supplement: S4 Table — (DOCX) [file pgen.1010666.s010.docx]

**Supplemental Table 4. Primary and secondary antibodies used for mouse chromatin spreads**

| **Antibody** | **Host** | **Source** | **Catalog Number** | **IF Dilution** |
| --- | --- | --- | --- | --- |
| ATR | Goat | Santa Cruz | sc-1887 | 1:50 |
| CDK2 | Mouse | Santa Cruz | sc-6248 | 1:50 |
| CYTIP | Rabbit | Novus | NBP1-88946 | 1:200 |
| RAD51 | Mouse | Invitrogen | MA5-14419 | 1:200 |
| RPA2 | Rabbit | Protein Tech Group | 10412-1-AP | 1:500 |
| SYCP1 | Rabbit | Thermo | PA1-16763 | 1:1000 |
| SYCP3 | Rabbit | Novus | NB300-231 | 1:1000 |
| SYCP3 | Goat | Novus Biologicals | AF3750 | 1:100 |
| SYCP3 | Mouse | Santa Cruz | sc-74568 | 1:50 |
| yH2AX | Mouse | Thermo | MA1-2022 | 1:1000 |
| Goat IgG (H+L) Alexa Fluor 488 | Donkey | Invitrogen | A-11055 | 1:500 |
| Goat IgG (H+L) Alexa Fluor 633 | Donkey | Invitrogen | A-21082 | 1:500 |
| Rabbit IgG (H+L) Alexa Fluor 568 | Donkey | Invitrogen | A-10042 | 1:500 |
| Mouse IgG (H+L) Alexa Fluor 488 | Donkey | Invitrogen | A-21202 | 1:500 |
| Mouse IgG (H+L) Alexa Fluor 568 | Donkey | Invitrogen | A-11031 | 1:500 |
| Rabbit IgG (H+L) Alexa Fluor 568 | Goat | Invitrogen | A-11011 | 1:500 |
| Mouse IgG (H+L), Alexa Fluor 488 | Goat | Invitrogen | A-11001 | 1:500 |
